# Younger age at diagnosis predisposes to mucosal recovery in celiac disease on a gluten-free diet: A meta-analysis

**DOI:** 10.1371/journal.pone.0187526

**Published:** 2017-11-02

**Authors:** Zsolt Szakács, Péter Mátrai, Péter Hegyi, Imre Szabó, Áron Vincze, Márta Balaskó, Bernadett Mosdósi, Patrícia Sarlós, Mária Simon, Katalin Márta, Alexandra Mikó, Dániel Pécsi, Alexandra Demcsák, Judit Bajor

**Affiliations:** 1 Institute for Translational Medicine, University of Pécs, Pécs, Hungary; 2 Institute of Bioanalysis, University of Pécs, Pécs, Hungary; 3 Hungarian Academy of Sciences-University of Szeged, Momentum Gastroenterology Multidisciplinary Research Group, Szeged, Hungary; 4 Division of Gastroenterology, 1st Department of Internal Medicine, University of Pécs, Pécs, Hungary; 5 Department of Paediatrics, University of Pécs, Pécs, Hungary; 6 Department of Psychiatry and Psychotherapy, University of Pécs, Pécs, Hungary; 7 Department of Pediatrics, University of Szeged, Szeged, Hungary; International Nutrition Inc, UNITED STATES

## Abstract

**Background and aims:**

Persistent intestinal damage is associated with higher complication rates in celiac disease. We aimed to assess the potential modifiers of mucosal recovery.

**Materials and methods:**

We screened databases (PubMed, Embase, Cochrane Trials, and Web of Science) for papers on celiac disease. Papers discussing (1) celiac patients (2) follow-up biopsy and (3) mucosal recovery after commencement of a gluten-free diet were included. The primary outcome was to produce a comprehensive analysis of complete mucosal recovery (i.e., Marsh 0 on follow-up). We compared children’s recovery ratios to those of adults. Patients following a strict gluten-free dietary regimen were included in a subgroup. Summary point estimates, 95% confidence intervals (CIs), and 95% predictive intervals (PIs) were calculated. Heterogeneity was tested with I^2^-statistic. The PROSPERO registration number is CRD42016053482.

**Results:**

The overall complete mucosal recovery ratio, calculated from 37 observational studies, was 0.36 (CI: 0.28–0.44, PI: -0.12–0.84; I^2^: 98.4%, p<0.01). Children showed higher complete mucosal recovery ratio than adults (p<0.01): 0.65 (CI: 0.44–0.85, PI: -0.10–1.39; I^2^: 96.5%, p<0.01) as opposed to 0.24 (CI: 0.15–0.33, PI: -0.19–1.08; I^2^: 96.3%, p<0.01). In the strict dietary adherence subgroup, complete mucosal recovery ratio was 0.47 (CI: 0.24–0.70, PI: -0.47–1.41; I^2^: 98.8%, p<0.001). On meta-regression, diagnostic villous atrophy (Marsh 3) ratio (-8.97, p<0.01) and male ratio (+6.04, p<0.01) proved to be a significant determinant of complete mucosal recovery, unlike duration of gluten-free diet (+0.01, p = 0.62). The correlation between complete mucosal recovery ratio and age on diagnosis is of borderline significance (-0.03, p = 0.05).

**Conclusions:**

There is considerable heterogeneity across studies concerning complete mucosal recovery ratios achieved by a gluten-free diet in celiac disease. Several celiac patients fail to achieve complete mucosal recovery even if a strict dietary regimen is followed. Younger age on diagnosis, less severe initial histologic damage and male gender predispose for achieving mucosal recovery.

## Introduction

Celiac disease is an immune-mediated systemic disorder. It can strike in any age in genetically susceptible individuals by consuming gluten-containing foods. The pathological reaction to gluten results in pathognomonic impairment of the small intestinal villous structure [1]. About 1% of the population in the United States and Western Europe is affected [2, 3].

In most patients in whom mucosal recovery cannot be achieved frequently [4], quick and dramatic improvement in symptoms is expected when switching to a gluten-free diet (GFD) [5]. Surprisingly, the intestinal mucosal recovery ratio ranges from 0 to 100% across studies [4, 6–68], the hypothesized modifiers are age on diagnosis [6–11, 13, 15, 16, 28, 42, 48, 51], duration of GFD [7, 9–14, 16, 25, 40, 42, 43, 51, 54], gender [6–13, 16, 69], initial histological severity [6–9, 11–15, 70], and dietary adherence [6–9, 11–13, 17, 28, 30].

The need for repeated biopsy is a matter of controversy: it is clearly recommended in patients remaining symptomatic on the long term GFD [71, 72]. However, symptoms [22, 37, 39, 73] and celiac-specific serology [74] correlate poorly with the follow-up histology (i.e., the degree of mucosal recovery), resulting in the misidentification of the non-recovered, who might be at higher risk of adverse outcomes in the long run [8, 9, 70, 73, 75, 76]. Intact mucosa has remained a desirable goal of the therapy.

To date, one meta-analysis related to mucosal recovery has been published, in which the focal question was the performance of celiac-specific antibody in predicting persistent villous atrophy, which resulted in a high exclusion rate of relevant articles [74]. Here, we planned to address the question whether celiac children on a GFD display higher mucosal recovery ratios than adults. In addition, the following potential modifiers of mucosal recovery were also examined: age on diagnosis, duration of and adherence to GFD, initial histological severity, and study quality. Our results might complement the careful selection process of subjects for whom a follow-up biopsy would be beneficial.

## Materials and methods

This meta-analysis was conducted and reported following the guidelines proposed by the Preferred Reporting Items for Systematic Reviews and Meta-Analyses (PRISMA) Statement [77] and the Meta-analysis Of Observational Studies in Epidemiology (MOOSE) Statement [78]. We registered the protocol *a priori* on PROSPERO under CRD42016053482 (S1 Appendix).

### Search

A manual search of the medical literature was performed in PubMed (MEDLINE), EMBASE, Cochrane Trials, and Web of Science from inception until Dec 30, 2016, for relevant articles that reported on mucosal recovery in celiac disease.

The PICO items of children-to-adults comparison were as follows: (P) celiac patients previously subjected to GFD with control biopsy, (I-C) adults and children, and (O) mucosal recovery ratios.

We used the free-text terms “celiac disease”, “mucosal healing”, “mucosal recovery”, and “villous atrophy”. Our search strategy was developed by using text words related to celiac disease by our review team of health care professionals and peer reviewed by an investigator with great expertise in systemic review searching. For the draft of our search strategy, see S2 Appendix.

The search was limited to human and English language studies in PubMed and Embase, to English language studies in Web of Science, but there were no filters imposed on the search in Cochrane Trials. A recursive hand search in each reference list of relevant and included articles was conducted to extend the coverage of the search.

PROSPERO, an international prospective register of systematic reviews, was hand searched for ongoing and completed meta-analyses.

### Selection and eligibility

The following publication types were excluded: letters, comments, conference abstracts, editorials, and reviews.

We included both experimental (randomized or non-randomized, controlled or uncontrolled clinical trials) and observational studies (cohort, cross-sectional and case-control studies) carried out either in a prospective or a retrospective manner without respect to the primary objectives of the studies. We excluded case reports and case series. The latest version of updates was included. If there were multiple publications from the same register, the most comprehensive report was included.

All the relevant articles were combined together in a reference manager software (EndNote X8) to remove duplicates by searching overlaps between titles, abstracts, authors, and publication years.

After having duplicates removed, review authors screened the articles by title, abstract, and full-texts against our pre-defined eligibility criteria. Each phase was carried out by two independent investigators in duplicate, none of whom were blinded to publication data. Third party arbitration resolved any discrepancies. We did not request any data from authors and did not incorporate unpublished material.

To be eligible, celiac patients were supposed to be subjected to GFD prior to the control biopsy which was staged by Marsh grades or any other histological classifications including only detailed text description of the mucosal status that can be converted into Marsh grades with minimal risk of bias [79–82]. Studies in which patients with childhood diagnosis (<18 years) were not separable from those with adulthood diagnosis (≥18 years) were excluded from the children-to-adults comparison but included in other subgroups and/or meta-regressions, when appropriate.

GFD is defined as the dietary exclusion of gluten-containing cereals (i.e., wheat, barley, and rye). The time elapsing between diagnostic and control biopsies is considered as the duration of prescribed GFD if not stated otherwise.

There is no consensus on the terminology of mucosal histologic recovery (S1 Table). Following previous research, we defined complete mucosal recovery (primary outcome) as Marsh 0 [6, 7, 12, 13, 15, 25, 53] and disappearance of villous atrophy (secondary outcome) as anything less than Marsh 3 (i.e., combined Marsh 0–2) [14, 22, 27, 39, 40, 45].

### Quality assessment

The Newcastle-Ottawa Scale (NOS) tool [83], dedicated to assessing cohort studies, was adjusted to the design of celiac studies (S2 Table). Items were assessed by one review author blinded to the publication data. On this scale, a study is judged by items with points (stars in the Newcastle-Ottawa Scale terminology) in three categories: selection of the study groups, comparability of the groups, and outcome of interest. We removed the comparability items due to the reasonable uncontrolled nature of celiac studies (i.e., endoscopic procedures would carry an excessive risk for healthy controls), thereby studies could award one star for each. Each item was rated as ‘high risk’ (equals to zero stars), ‘low risk’ (equals to one star), or ‘unclear risk’ (equals to zero stars) corresponding to the definitions (S2 Table). At the end, we calculated the overall methodological quality of each study by adding the stars (a maximum of six stars could be awarded). Overall quality scores were incorporated in the statistical analysis. More than four scores indicated high methodological quality.

We used Grading of Recommendations Assessment, Development and Evaluation (GRADE) [84] methodology for rating the quality of evidence as very low, low, moderate, or high.

Studies recruiting patients on GFD before the control biopsy were labeled as prospective, while all the other ones were considered as retrospective.

### Data extraction

Data were extracted in duplicates by two independent investigators onto a standardized form designed *a priori*. If needed, data were approximated from figures and graphs. Third party arbitration resolved any discrepancies. Numeric and texted data were collected, as listed in S2 Appendix.

### Statistical analysis

The statistical analysis was completed by a trained biostatistician expert (PM) by means of Comprehensive Meta-analysis Software (Version 3, Biostat, Englewood) and Stata 11 SE (Stata Corp). Recovery ratios were pooled using the random effects model with the DerSimonian-Laird estimation and displayed on forest plots. Summary point estimations, 95% Confidence Intervals (CIs), and 95% Predictive Intervals (PIs) were calculated from recovery ratios.

Statistical heterogeneity was tested using the I^2^ statistic adapting the thresholds of the Cochrane Handbook of Systemic Reviews of Interventions: 0–40%, 30–60%, 50–90%, and 75–100% indicated not important, moderate, substantial, and considerable heterogeneity [85]. Chi-square test was used for gaining probability-values (p<0.01 indicated significant heterogeneity) [86].

To explore heterogeneity, we performed subgroup analyses and univariate meta-regressions. When different groups were compared, p<0.05 indicated a significant difference, while we took 0.10>p≥0.05 as borderline significance. Comparisons of mucosal recovery ratios were made as follows: (1) children to adults, (2) prospective to retrospective studies (3) patients with strict GFD to those with non-strict or unknown adherence, (4) strict adherence to at least 12-month gluten-free diet to others, (5) patients followed up for 12 months to others, (6) recovery ratios assessed by Marsh to Marsh-Oberhuber classification, different risk groups (by S2 Table) concerning (7) dietary assessment, (8) duration of GFD, and (9) diagnostic histology; the latter was performed only with the secondary outcome. In meta-regressions, the outcome variable was Marsh 0 or 0–2 ratio; the explanatory variable was mean (first-preference) or median of age on diagnosis in years or duration of GFD in months, initial Marsh 3 ratio, male ratio, or methodological quality (overall quality score). We report the number of studies included in the model, the regression coefficient, and the corresponding p-value on each meta-regression where p<0.05 indicated a significant linear association.

Sensitivity analysis was used to assess the impact of high-risk studies by omitting them from the analyses and recalculating to investigate their effects on the overall estimation.

The small-study effect was tested by the Egger’s test [87], p<0.05 indicated proof of bias.

Cohen’s Κ was calculated for measuring agreement between the investigators in each phase of selection. Values of kappa statistics can be interpreted, as follows: ≤0 as declaring no agreement, 0.01–0.20 as slight, 0.21–0.40 as fair, 0.41–0.60 as moderate, 0.61–0.80 as substantial, and 0.81–1.00 as nearly perfect agreement [88].

## Results

**Fig 1:**
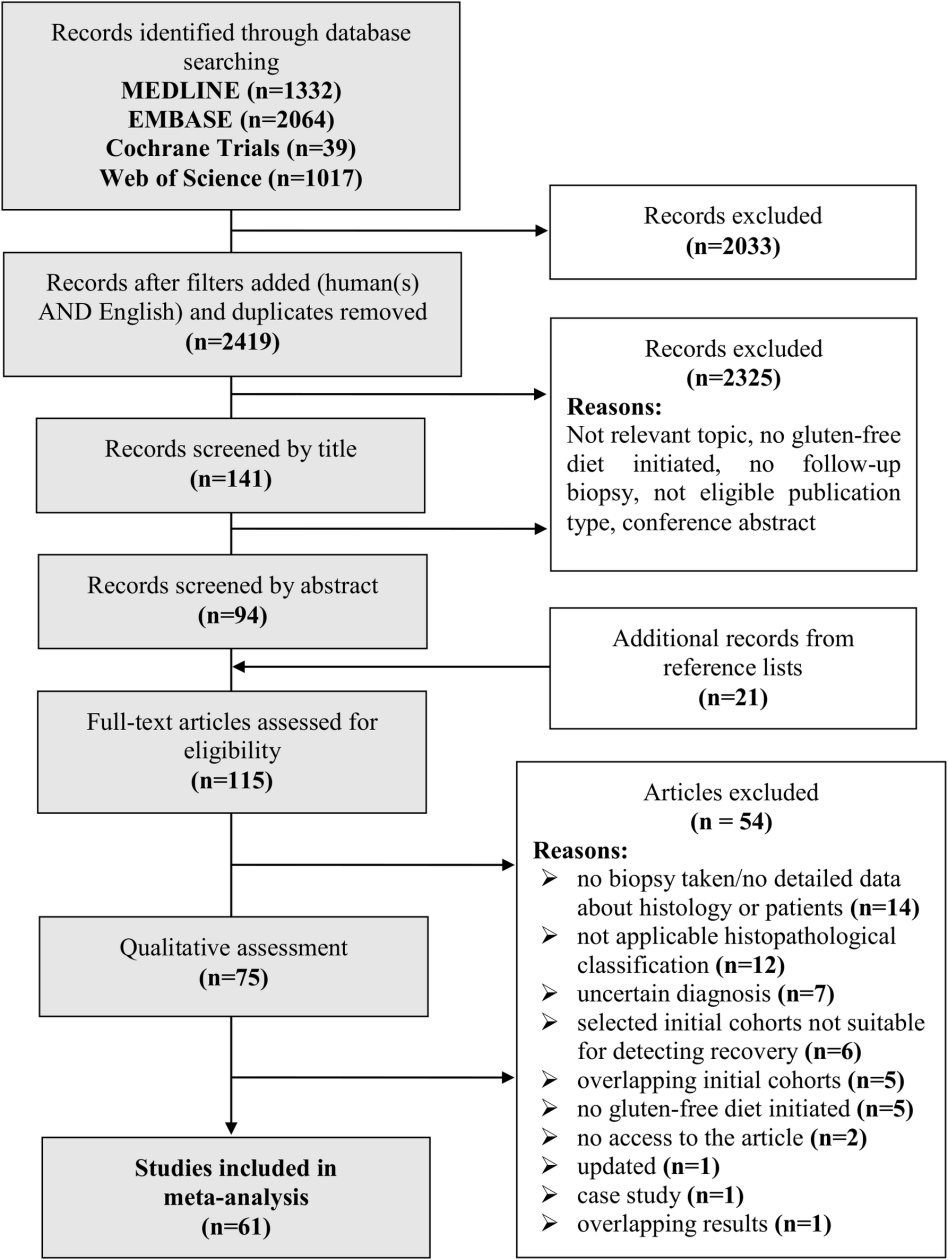
Flow chart of assessment of studies identified in the meta-analysis.

Fig 1 shows the flowchart of this meta-analysis. Our search strategy yielded 4452 studies of which we selected 94 for full-text assessment and added another 21 from reference lists. Finally, 61 studies met our inclusion criteria, none of them was a randomized-controlled trial (Table 1, S3 Table) [4, 6–34, 36–55]. Only one [36] of six articles [36, 69, 70, 75, 89, 90] with identical initial cohorts was included in full-text assessment (S4 Table). Not given the data on recovery in ineligible formats, we excluded twelve articles [35, 91–101] after quality assessment. Cohen’s К was 0.78, 0.79, and 0.87 for selection by title, abstract, and full-text, respectively; indicating at least substantial agreement between the investigators in each phase of selection.

**Table 1:** Characteristics of the included studies. H: high quality, L: low quality, NR: not reported, P: prospective, R: retrospective.

Study	Country	Recruitment period	Design	Histological Classification
Annibale, 2001 [29]	Italy	1994–1997	P	Modified Marsh
Assiri, 2008 [44]	Saudi Arabia	10 years	R	NR
Bannister, 2014 [45]	Australia	2009–2011	R	Marsh-Oberhuber
Bardella, 2007 [10]	Italy	NR	R	Marsh-Oberhuber
Baudon, 2005 [46]	France	1971–1982	P	NR
Bhasin, 2010 [15]	India	NR	P	Marsh
Biagi, 2012 [30]	Italy	2008–2011	P	After O'Mahony
Cammarota, 2007 [31]	Italy	2004–2005	P	Marsh-Oberhuber
Capristo, 2009 [56]	Italy	1996–2007	P	Marsh-Oberhuber
Carroccio, 2008 [20]	Italy	2005–2006	P	Marsh-Oberhuber
Caruso, 2014 [18]	Italy	2011–2013	P	Corazza-Vilanacci
Casella, 2012 [48]	Italy	1990–2010	R	Marsh-Oberhuber
Chaisemartin, 2015 [57]	United Kingdom	2008–2012	R	Marsh-Oberhuber
Ciacci, 2002 [11]	Italy	until 1997	R	Marsh-Oberhuber
Ciacci, 2005 [47]	Italy	NR	P	NR
Congdon, 1981 [49]	United Kingdom	NR	P	Reported text description
Cuoco, 1998 [62]	Italy	1993–1996	P	Reported text description
Dickey, 2000 [21]	United Kingdom	1996–1998	P	Marsh-Rostami
Donaldson, 2008 [58]	United States	NR	R	Marsh-Oberhuber
Duerksen, 2010 [63]	Canada	NR	P	Modified Marsh
Elli, 2015 [23]	Italy	2000–2012	R	Marsh-Oberhuber
Galli, 2014 [6]	Italy	2009–2012	P	Marsh-Oberhuber
Ghazzawi, 2014 [50]	United States	1997–2013	R	Modified Marsh
Gorgun, 2009 [34]	Belarus	NR	P	Marsh
Günther, 2010 [59]	Germany	2007–2009	P	Marsh-Oberhuber
Hære, 2016 [22]	Norway	1989–2009	R	Marsh-Oberhuber
Hopper, 2008 [24]	United Kingdom	2004–2006	P	Marsh-Oberhuber
Hutchinson, 2010 [13]	United Kingdom	from 1971	R	Modified Marsh
Karinen, 2006 [19]	Finland	NR	P	Reported text description
Kaukinen, 2002 [38]	Finland	NR	P	Marsh
Kemppainen, 1998 [52]	Finland	1988–1990	P	Reported text description
Koskinen, 2010 [60]	Finland	NR	P	Reported text description
Lanzini, 2009 [7]	Italy	from 1990 on	R	Marsh and Marsh-Oberhuber
Lebwohl, 2013 [36]	Sweden	1969–2008	R	Marsh
Lee, 2003 [5]	United States	NR	R	Reported text description
Lichtwark, 2014 [25]	Australia	NR	P	Marsh
Lidums, 2011 [39]	Australia	2006–2009	P	Marsh-Oberhuber
Martini, 2002 [53]	Italy	2000–2001	P	Marsh
McMillan, 2001 [26]	United Kingdom	NR	P	Marsh-Rostami
Newnham, 2016 [12]	Australia	NR	P	Marsh
O’Keeffe, 2001 [64]	Ireland	NR	P	NR
Pekki, 2015 [8]	Finland	1996–2009	P	Reported text description
Raivio, 2006 [65]	Finland and Hungary	NR	P	Reported text desciption
Rubio-Tapia, 2010 [9]	United States	until 2008	R	Marsh-Oberhuber
Selby, 1999 [16]	Australia	1994–1997	P	Reported text description
Sharkey, 2013 [40]	United Kingdom	until 2012	R	Marsh-Oberhuber
Shmerling, 1986 [41]	Switzerland	1960–1983	R	After Shmerling
Sjöberg, 2014 [66]	Sweden	1998–2002	R	Marsh
Tuire, 2012 [37]	Finland	NR	P	Marsh
Tursi, 2006 [42]	Italy	2001–2004	P	Marsh
Uil, 1996 [54]	The Netherlands	NR	P	Reported text description
Vahedi, 2003 [17]	France	1994–1999	P	Marsh-Oberhuber
Valdimarsson, 2000 [55]	Sweden	1989–1997	P	Alexander
Vécsei, 2009 [28]	Austria	1989–2006	R	Marsh-Oberhuber
Vécsei, 2014 [51]	Austria	2009–2010	P	Marsh-Oberhuber
Vivas, 2009 [27]	Spain	2000–2008	P	Marsh-Oberhuber
Volta, 2008 [67]	Italy	2005–2006	P	Marsh-Oberhuber
Wahab, 2001 [61]	The Netherlands	NR	P	Marsh-Rostami
Wahab, 2002 [14]	The Netherlands	1985–2000	R	Marsh
Yachha, 2007 [43]	India	1991–1999	P	Marsh
Zanini, 2012 [68]	Italy	2001–2010	R	Marsh

### Pooled effects

**Fig 2:**
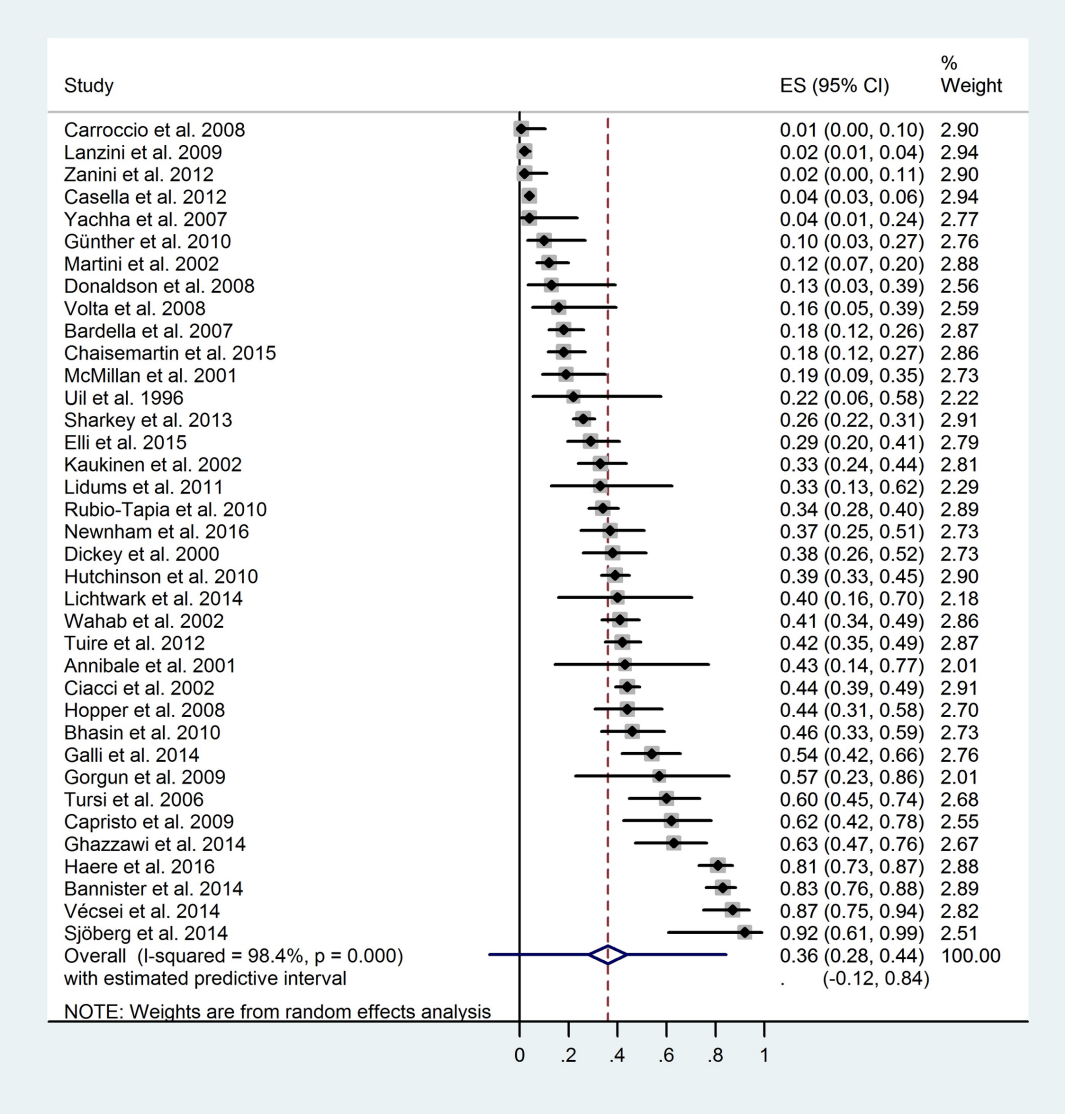
Forest plot: Complete mucosal recovery ratios of each study included.

Here, we pooled the histologic recovery ratios of small intestinal mucosa. The pooled complete mucosal recovery ratio, calculated from 37 studies, was 0.36 (CI: 0.28–0.44, PI: -0.12–0.84; I^2^: 98.4%, p<0.01) (Fig 2). The pooled disappearance of villous atrophy ratio, calculated from 57 studies, was 0.64 (CI 0.58–0.70, PI: 0.23–1.05; I^2^: 97.5% p<0.01) (S1 File).

### Subgroups and meta-regressions

**Fig 3:**
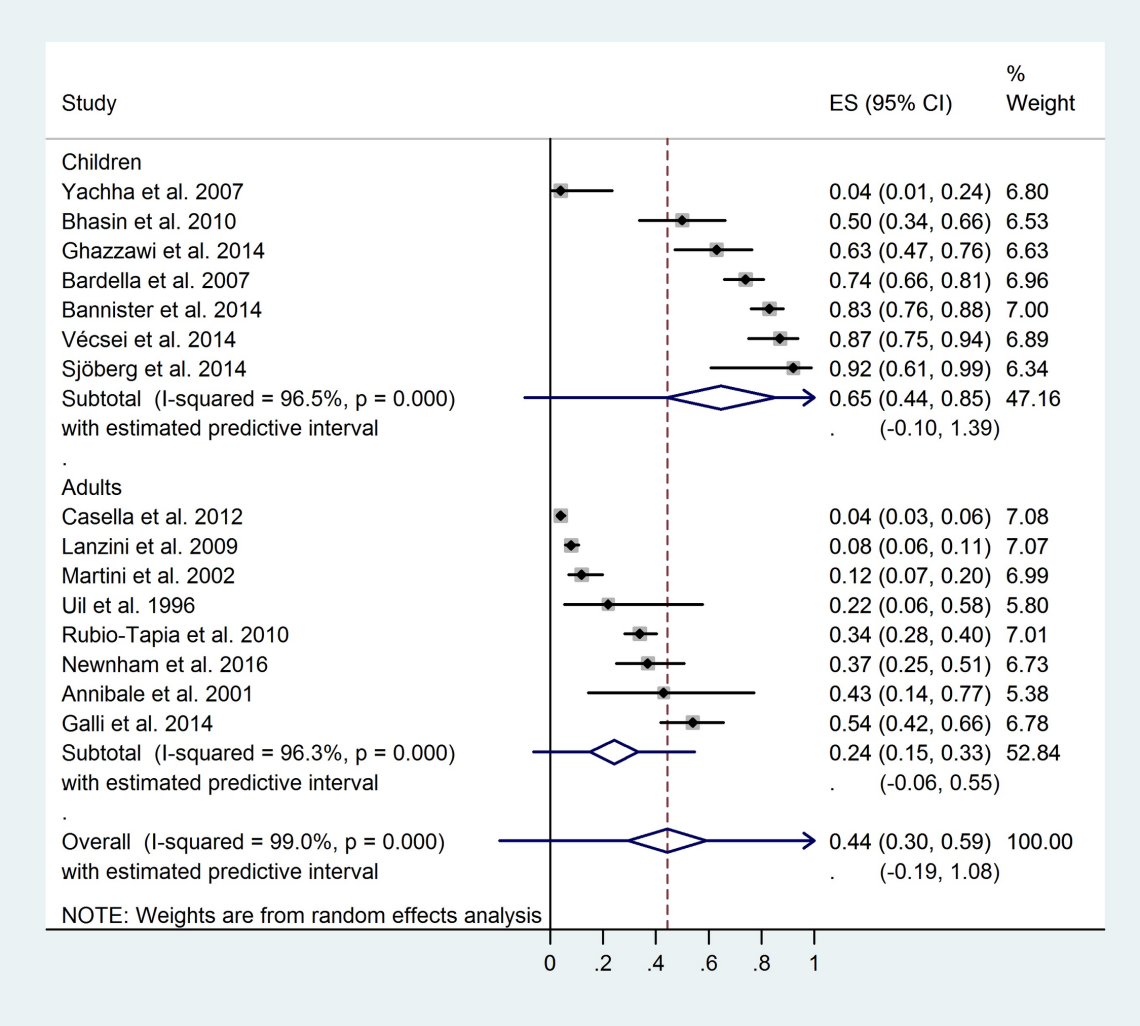
Forest plot: Complete mucosal recovery ratios of children-to-adults comparison.

Children showed higher control Marsh 0 ratio: 0.65 (CI: 0.44–0.85, PI: -0.10–1.39; I^2^: 96.5%, p<0.01) vs. 0.24 (CI: 0.15–0.33, PI:-0.19–1.08; I^2^: 96.3%, p<0.01), p<0.01 (Fig 3). Similar difference with borderline significance was observed concerning the control Marsh 0–2 ratio (S1 File).

In the subgroup of strict dietary adherence, control Marsh 0 ratio was only 0.47 (S2 File) and control Marsh 0–2 ratio was 0.72 (S1 File). We gained similar recovery ratios when we included only patients with good dietary adherence and at least 12-month follow-up: 0.44 and 0.77 for control Marsh 0 and 0–2, respectively.

**Fig 4:**
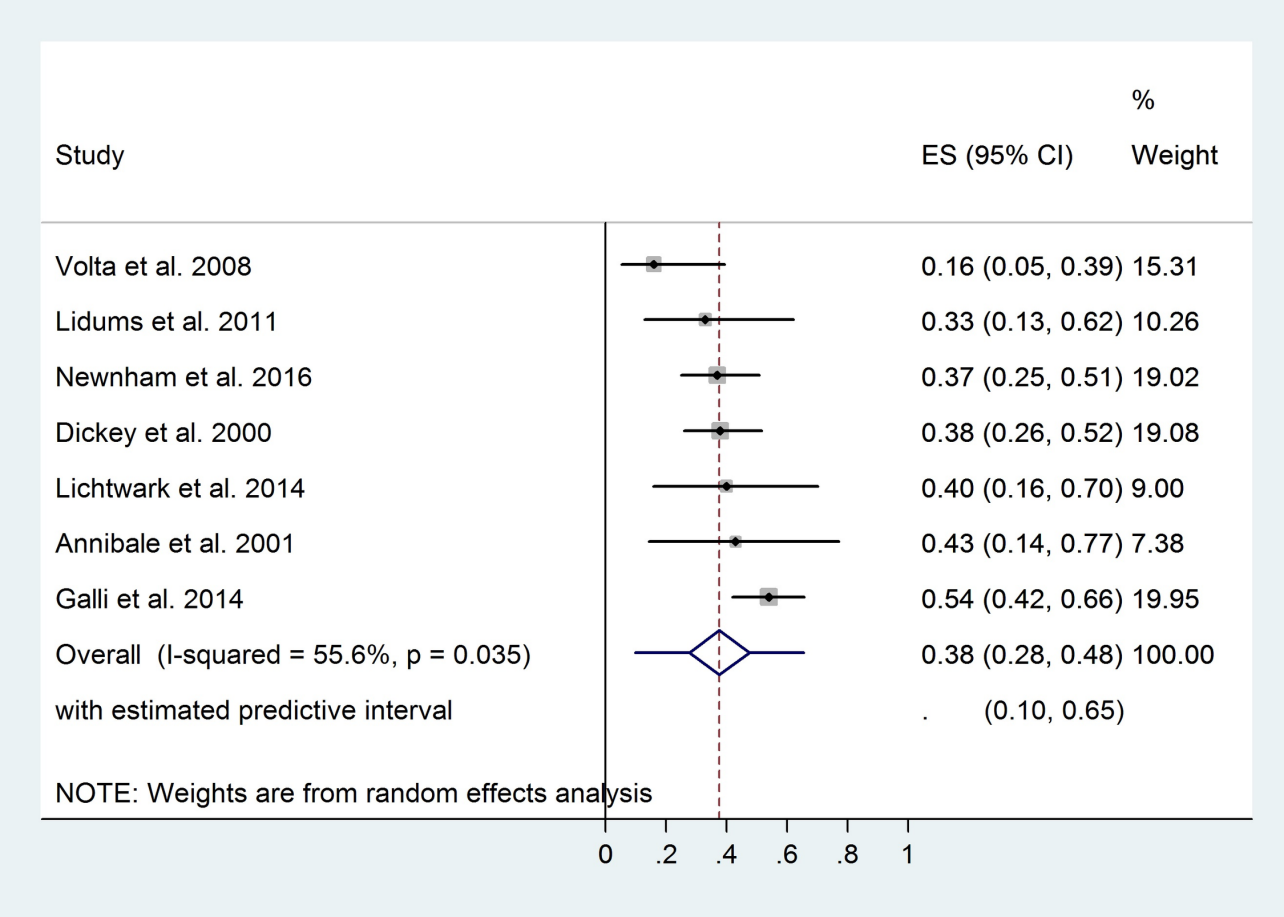
Forest plot: Complete mucosal recovery ratios of patients after 12-month gluten-free diet.

At the 12^th^ month of gluten-free diet, only 38% (Fig 4) and 54% (S1 File) of patients achieved complete mucosal recovery and disappearance of villous atrophy, respectively.

The control Marsh 0 and 0–2 ratios calculated by Marsh or Marsh-Oberhuber did not differ significantly (p>0.10). Neither the study design (prospective vs. retrospective) nor the method used for assessing dietary adherence affected the recovery ratios (p>0.10). Control Marsh 0–2 ratio was higher in studies including only patients with villous atrophy on diagnosis, as compared to inclusion of patients with lesser histologic damage. However, the level of significance was only borderline (p = 0.09).

I^2^ test showed considerable heterogeneity (except for the subgroup of patients with 12-month of diet with moderate heterogeneity), which proved to be significant in and across the subgroups (p<0.01). Detailed results of the analyses are given in Table 2.

**Table 2:** Results of subgroup analyses.

	p	Recovery ratios	Number of studies (n)	I^2^ across groups
Complete mucosal recovery				
Children vs. adults	<0.01*	0.65 (0.44–0.85) vs. 0.24 (0.15–0.33)	7 vs. 8	99.0%
Strict vs. non-strict/uncertain gluten-free diet	0.39	0.47 (0.24–0.70) vs. 0.33 (0.23–0.45)	12 vs. 26	95.6%
Strict adherence with at least 12-month gluten-free diet vs. others	0.32	0.44 (0.26–0.64) vs. 0.33 (0.23–0.44)	10 vs. 27	95.7%
Patients followed up for 12 months vs. others	0.70	0.38 (0.28–0.48) vs. 0.36 (0.27–0.45)	7 vs. 30	98.3%
Length of gluten-free diet (high vs. low risk)	0.91	0.34 (0.20–0.52) vs. 0.33 (0.24–0.44)	10 vs. 27	95.0%
Assessment of adherence (high vs. low vs. uncertain risk)	0.25	0.62 (0.27–0.88) vs. 0.33 (0.23–0.44) vs. 0.29 (0.17–0.45)	3 vs. 22 vs. 12	95.1%
Marsh vs. Marsh-Oberhuber classification	0.76	0.31 (0.18–0.47) vs. 0.34 (0.22–0.47)	13 vs. 18	95.7%
Prospective vs. retrospective design	0.86	0.34 (0.23–0.48) vs. 0.33 (0.20–0.47)	19 vs. 14	95.4%
**Disappearance of villous atrophy**				
Children vs. adults	0.05^#^	0.74 (0.57–0.90) vs. 0.58 (0.45–0.71)	11 vs. 10	96.5%
Strict vs. non-strict/uncertain gluten-free diet	0.06^#^	0.72 (0.60–0.84) vs. 0.66 (0.59–0.72)	17 vs. 40	92.8%
Strict adherence with at least 12-month gluten-free diet vs. others	0.08^#^	0.77 (0.67–0.85) vs. 0.66 (0.60–0.72)	14 vs. 43	92.9%
Patients followed up for 12 months vs. others	0.03*	0.54 (0.41–0.67) vs. 0.67 (0.60–0.73)	12 vs. 45	97.5%
Length of gluten-free diet (high vs. low vs. uncertain risk)	0.68	0.70 (0.56–0.81) vs. 0.65 (0.59–0.71) vs. 0.58 (0.34–0.79)	10 vs. 44 vs. 3	92.9%
Assessment of adherence (high vs. low vs. uncertain risk)	0.60	0.59 (0.33–0.81) vs. 0.64 (0.56–0.71) vs. 0.69 (0.60–0.76)	4 vs. 27 vs. 26	92.9%
Initial histology (high vs. low vs. uncertain risk)	0.09^#^	0.73 (0.64–0.80) vs. 0.61 (0.54–0.65) vs. 0.70 (0.58–0.80)	12 vs. 32 vs. 9	92.9%
Marsh vs. Marsh-Oberhuber classification	0.39	0.71 (0.59–0.81) vs. 0.65 (0.55–0.73)	12 vs. 21	94.6%
Prospective vs. retrospective design	0.13	0.63 (0.56;0.70) vs. 0.72 (0.63–0.79)	36 vs. 17	92.9%

* indicates statistical significance (p<0.05).

^#^ indicates borderline significance (0.10>p≥0.05).

Age on diagnosis showed a significant negative linear correlation with control Marsh 0–2 ratio (p<0.01) but we observed only a tendency concerning Marsh 0 ratio (p = 0.05). Interestingly, the duration of gluten-free diet did not affect recovery ratios (the correlation coefficient was 0). Having omitted the studies with extreme length of follow-up from the analyses (more than about 2 years of diet), we found a non-significant positive correlation (p = 0.13 and p = 0.18 for control Marsh 0 and 0–2 ratios, respectively). Diagnostic Marsh 3 ratio was closely associated with control Marsh 0 and 0–2 ratios (p<0.01), here we observed a strong negative linear correlation (coefficient: -8.97 and -12.09 for control Marsh 0 and 0–2 ratios, respectively. Male sex ratio was clearly associated with higher control Marsh 0 ratio (p<0.01) but not with control Marsh 0–2 (p = 0.87). Detailed results and figures of the analyses are given in Table 3 and in S3 File, respectively.

**Table 3:** Detailed results of meta-regressions.

	p	Coefficient	Number of studies
**Complete mucosal recovery**			
Age at diagnosis	0.05^#^	-0.03	18
Length of gluten-free diet	0.62	0.01	21
Length of gluten-free diet (9–26.4 months)	0.13	0.09	16
Diagnostic Marsh 3 ratio	<0.01*	-8.97	17
Male ratio	<0.01*	6.04	29
Quality	0.08^#^	0.28	33
**Disappearance of villous atrophy**			
Age at diagnosis	<0.01*	-0.03	24
Length of gluten-free diet	0.86	0.00	33
Length of gluten-free diet (9–27 months)	0.18	0.04	17
Diagnostic Marsh 3 ratio	<0.01*	-12.09	17
Male ratio	0.87	0.24	42
Quality	0.85	0.02	53

* indicates statistical significance (p<0.05).

^#^ indicates borderline significance (0.10>p≥0.05).

### Quality of evidence

This meta-analysis included observational studies of which 21 (34%) were retrospective and most of them were uncontrolled (S5 Table). We rated the quality of evidence as very low due to risk of bias, inconsistency and the high number of uncontrolled studies.

### Sensitivity analysis

The removal of high-risk articles [13, 36] did not influence statistical significance.

### Small-study effect

We could not prove the presence of small-study effect (p = 0.93 and p = 0.11 for pooled control Marsh 0 and 0–2 outcomes, respectively) (S4 File).

## Discussion

Here, we aimed to investigate histologic recovery ratios of small intestinal mucosa in celiac patients subjected to GFD, with special emphasis on the modifying effect of age on diagnosis. Our findings are consistent with the previous meta-analysis, where persistent villous atrophy was detectable in about one third (38%) of the celiac population and children tended to have lower atrophy ratios (19%), as compared to adults (38%) [74]. Here, we confirmed that persistent villous atrophy ratio is inexplicably common in treated celiac patients (in about one third of the patients), so are persistent mucosal abnormalities (in about two third of the patients). Consequently, two third of the patients had intact villous architecture (S1 File) and one third of them achieved complete mucosal recovery (Fig 2). We also found that childhood diagnosis was closely associated with higher complete recovery ratios (65% vs. 24% and 74% vs. 58% for complete recovery (Fig 3) and disappearance of villous atrophy (S1 File)). The favorable impact of early age at diagnosis was also confirmed with regression analysis (Table 3 and S3 File).

As we expected, statistics revealed considerable heterogeneity and consequent wide predictive intervals (Table 2), which reflects differences in study settings, baseline characteristics, follow-up times, dietary adherence, and methodology (S6 and S7 Tables).

### The exploration of heterogeneity

Besides good dietary adherence [6, 7, 9, 11, 13, 33], other modifiers of mucosal recovery are less clear. Poor adherence can be the cause of persistent symptoms [6]; furthermore, lack of GFD leads to increased mortality [102, 103]. However, poor adherence cannot explain the low recovery ratios itself and good adherence cannot guarantee high ratios [9]. Our results are in line with this finding: only 47% of strictly adherent patients achieved complete mucosal recovery (S2 File) and 72% had intact villi (S1 File). Recovery ratios might have been underestimated due to short follow-up; however, including only studies with at least 12-month of gluten-free diet did not improve the recovery ratios considerably (±5%) (Table 2). Interestingly, less than 50% of patients achieved complete mucosal recovery in seven studies with good adherence [12, 20, 21, 25, 37, 38, 43] in two studies [20, 43], complete recovery (i.e., Marsh 0) was not achieved almost at all, as well as disappearance of villous atrophy ratio (i.e., Marsh 0–2) was surprisingly low in three studies [20, 21, 43]. In one cohort including only children [43], 5 years of strict GFD was not enough to achieve Marsh 0 but it was enough to downgrade the histologic damage to Marsh 1–2. Here we question the strictness of the diet; besides, infections, delayed diagnosis, and genetic background are hypothesized as impeding factors of recovery. Similarly, another author [21] could not show higher than 40% of complete recovery ratio within 12-month follow-up, despite good adherence. In one study [20], poor recovery ratios might be explained by selection bias: patients with refractory gastrointestinal symptoms comprised the majority of the study population. Turning to the other end of the spectrum, almost every patient (>90%) reached complete recovery on a strict diet [11]. Although it was a long follow-up study (2–22 years), they could not establish a significant correlation between the duration of follow-up and mucosal recovery. Despite the high recovery ratio, a negative correlation existed between the follow-up time and the dietary adherence [11].

In children diagnosed over four years of age, dietary adherence dropped [104] while the recovery ratio might have dropped in parallel with it although separate data on children are not available [30]. Consequently, it seems reasonable to belive that the earlier celiac disease is diagnosed, the better recovery ratio can be achieved in the long run. It is possible that this drop in dietary adherence with aging is responsible for the observed negative correlation between recovery ratios and age on diagnosis. In adults, literature results are nearly consistent: a strong correlation between the ratio of incomplete recovery and poor adherence was frequently detected [6, 8, 9, 11–13, 17, 33]. As to the cause of various recovery ratios, we cannot rule out inadvertent gluten ingestion, even in those who adhered to a strict GFD [105, 106] but persisting atrophy is unlikely to be the consequence of mild dietary transgressions (Codex-GFD) [16]. Foods containing trace amounts of gluten (<50 mg/day, i.e., occult sources or gluten contamination) are considered relatively safe [32]. Intraepithelial lymphocytosis reduction—and especially that of γδ T-cells—requires a longer period of time without dietary transgressions as compared to the resolution of villous atrophy [107, 108] and might contribute to the unexpectedly low control Marsh 0 ratios (Fig 2).

The beneficial effect of early age on diagnosis is a matter of controversy. In general, children tend to recover faster than adults [10, 14, 15]. Young age on diagnosis (*i*.*e*. an early diagnosis) was found an independent predictor of recovery [10], while other findings did not confirm this [6–8, 12]. Each year delay in the diagnosis results in an extra 1.106 odds ratio of having persisting duodenal abnormalities (2.751 odds ratio for 10 years) [10]. We confirmed an inverse correlation between the age on diagnosis (under 50 years) and control Marsh 0–2 ratio in regression analysis (p<0.01) and a tendency with control Marsh 0 (p = 0.05) (S3 File). Again, better adherence in childhood can be a reasonable confounding factor [104]. The correlation might reflect the cumulative (lifetime) gluten exposure, but in general, the duration of gluten exposure is proportional to age on diagnosis and to the disease duration, consequently. Taken together, the earlier the diagnosis is made the higher the chance of achieving complete mucosal recovery thereby avoiding the sequelae of persisting damage.

According to the American College of Gastroenterology (2013), control biopsies should be taken after 2 years of GFD from adults and not routinely from children in order to assess mucosal recovery [72]. Nevertheless, long-term follow-up studies of high quality are lacking. We do not assume a positive correlation between duration of GFD and recovery ratios, not even within about two years of diet (S3 File). We could separate neither children from adults nor patients with different dietary adherence in regression analysis due to the low amount of corresponding data. These findings are in accordance with a previous study, where the length of GFD did not predict mucosal recovery in the long run (2–22 years) [11]. Lack of correlation might imply no further (or just negligible) improvement in mucosal status. Therefore, taking control biopsy from symptomless patients repeatedly seems to be unnecessary because intestinal mucosa might not tend to change in the long run. As a matter of fact, the present analysis suffers from the limitation that we did not have data about mucosal status within 9 months of gluten-free diet which might be the critical period of the development of mucosal recovery. The 12^th^ month of the diet is a widely accepted time point to assess mucosal recovery. Although control Marsh 0 ratio is similar to the pooled effect (Fig 4), control Marsh 0–2 ratio seems to be lower (S1 File), suggesting further recovery. Control biopsy should be scheduled for later than this time point to improve the identification rate of those with true persistent villous atrophy.

Theoretically, it is plausible that the speed of recovery varies among people hence it is hard to distinguish those with slow, gradual improvement from those with persisting damage without taking repeated samples. Initial presentation, such as classical celiac disease [109] or malabsorption [8], might require longer time to recover, probably due to more severe initial damage. It is also possible that mucosal recovery occurs periodically in accordance with the intensity of the spontaneous or gluten-triggered local inflammation. The mucosal expression of genes activated in Th1 response (e.g., *STAT1*, *IRF1*), remained still enhanced, and was accompanied by a suppressed Th2 response, after 1 year of GFD [110]. Low recovery ratios (despite a long, strict GFD) might be explained by this gluten-independent immune dysregulation and persisting inflammation.

The assumption that more severe initial histology permits less/slower histological response is well supported by a large body of evidence [6–9, 13–15, 69]. Three studies [6, 7, 69] found initial histological severity an independent predictor of recovery, and only two studies [11, 12] did not support this. Our results are congruent with the general opinion: the initial Marsh 3 ratio correlates inversely with the frequency of control Marsh 0 and 0–2 ratios (S3 File). Here, the possible confounding effect of age and dietary adherence was not taken into account.

Male gender seems to be a predictor of mucosal recovery. The positive correlation was significant in terms of control Marsh 0 (S3 File). Most previous research considered the gender neutral [6–11, 13, 23], while two studies found it as an independent predictor recovery [7, 12]. Our results opposed the largest cohort so far [69] where female gender was associated with mucosal recovery. Gender-dependent difference in dietary adherence is unlikely to be a confounding factor since males proved to be as adherent as females earlier [111].

As a matter of fact, the items of the adapted Newcastle-Ottawa Scale did not cover the entire sampling, processing, and evaluating procedure. In addition, detailed descriptions of sampling methods were scarce in our material: half of the studies included did not publish sufficient information about the endoscopic procedure, biopsy sampling, and histological preparations. Furthermore, methodological discrepancies were common and likely to contribute to the considerable heterogeneity (S6 and S7 Tables).

### Prognostic role of mucosal recovery

Although persisting intestinal damage often co-occurs with higher frequency of comorbidities (e.g., metabolic osteopathy [8, 70, 73, 76] or malignancies [9, 73, 75]), recent evidence suggests that lack of mucosal recovery is not associated with higher mortality in the long run [8, 36].

### Strengths and limitations

The main strength of this meta-analysis is its comprehensiveness. Although we faced significant heterogeneity, various possible sources were explored, which resulted in convincing explanations. Despite the low case numbers of the individual studies, we could not prove significant small-study effect. The funnel plots are symmetric (S4 File). The methodological quality of the studies included was rigorously assessed (S2 and S5 Tables) and incorporated in the synthesis (Table 3).

It has to be mentioned that only observational studies fulfilled our eligibility criteria: to date, no randomized-controlled study has been published focusing on mucosal recovery. Although uncontrolled, retrospective study design was common, the comparison of prospective and retrospective studies did not yield a significant difference (Table 2). The quality of evidence (GRADE) was rated very low for both outcomes [84]; however, low quality is inherent in meta-analysis of observational studies [85].

Alternative variables falling outside the scope of this piece of work should be contemplated: e.g., study setting, various endoscopic and sampling protocols, difference in sample preparation or in the fashion of histological assessment. All of these factors might contribute to heterogeneity (S6 Table). So does genetic background: although one study [19] reported a correlation between *HLA-DQ2* gene dose and recovery ratios, this was not confirmed later [7]. The effects of other loci on histological recovery are unknown. The presence of *Helicobacter pylori* infection is unlikely to impede mucosal recovery but might be the cause of persisting intraepithelial lymphocytosis [7, 10]. Higher levels of patients’ background knowledge associated with the better histological response can also be a confounding factor [11, 69].

The dietary interview carried out by a trained interviewer has still remained the best tool: it predicts the persisting damage with proper accuracy. Discrepancies in dietary assessment across studies were tremendous, many did not even report on how they assessed GFD, raising doubts about the true dietary adherence (S7 Table). We must mention that there is an urgent need for the development of an accurate and objective method to assess true dietary adherence. Based on the results of the last decades, the pervasive celiac-specific serology exhibited low specificity in detecting persisting mucosal damage [74]. Short diet can distort the results, but the recovery ratios of the studies including patients with a GFD shorter than 1 year did not differ significantly from the longer ones (Table 2).

The inclusion of those not having villous atrophy on diagnosis might distort the calculations of Marsh 0–2 outcome (the outcome might be present before the diet). We dismissed this hypothesis because the recovery ratio proved to be even higher in the subgroup of high-risk studies, as compared to low-risk ones (Table 2).

Conversion of histological classifications into Marsh grades can lead to mild distortions in the set of data [112]. The cut-off between normal and pathological intraepithelial lymphocytosis ranges between 25 and 40 per 100 enterocytes [80–82] in between the change of the cut-off does hardly introduce bias [7]. We could not detect significant difference between the recovery rates graded by Marsh or Marsh-Oberhuber classification (Table 2).

Non-English language articles were excluded due to the lack of resources available to translate articles, raising the possibility of missing relevant articles.

Data on age and duration of GFD were given as mean and/or median due to inconsistent follow-up periods. Having assumed these values to be representative of the initial sample, we used them as explanatory variables in meta-regressions.

## Conclusions

Despite the prescribed GFD, we found unexpectedly low complete mucosal recovery ratios (36%), even in patients being on a strict diet (47%). Disappearance of villous atrophy was more frequent (64%) than complete recovery, but far from 100% even in those following a strict diet (72%). These numbers emphasize the importance of performing a control biopsy in celiac patients to detect persisting histologic damage predisposing to several adverse outcomes (e.g., osteoporosis, malignancies).

In a 12-month gluten-free diet, 38% and 54% of patients exhibited complete mucosal recovery and disappearance of villous atrophy, respectively. However, one year of diet might be insufficient to achieve mucosal recovery therefore biopsies should be taken later.

Children show higher complete recovery (65%) and disappearance of villous atrophy ratios (74%) as compared to adults (24% and 58%, respectively), which was supported with regression analysis. Early diagnosis (therefore early initiation of the diet) can lead to higher mucosal recovery ratios; consequently, early diagnosis might help to reduce the frequency of adverse outcomes in the long run. This conclusion should be confirmed by prospective studies.

We could not confirm the positive correlation between the length of GFD and mucosal recovery ratios either in the long run or between 9 months and about 2 years of diet. Accordingly, repeated control biopsies should not be taken from asymptomatic patients compliant with the diet to monitor mucosal recovery because results do not imply further histological improvement after several years of GFD.

Regression analysis revealed that more severe diagnostic histologic damage and female gender is closely associated with low mucosal recovery ratios. These risk groups might require stricter follow-up and GFD.

Given the considerable heterogeneity of the included studies, we would like to encourage the authors to follow the standard endoscopic and histologic procedures when conducting research in the field.

Our results might contribute to the identification of those subjects who would benefit from a control biopsy. Well-designed, controlled studies with large sample size are needed to validate our findings and discover further associations.

## Supporting information

S1 Appendix Prospero registration. (PDF)

S2 Appendix Search draft and list of collected data. (PDF)

S1 Table Different approaches to the definition of mucosal recovery in the literature. (DOCX)

S2 Table Items of the adapted Newcastle-Ottawa Scale. (DOCX)

S3 Table Data used in statistical analysis. NR, not reported; *, mean; ^#^, median; ^s^, same follow-up period for all participants.
(DOCX)

S4 Table Rationales for exclusion on full-text assessment. (DOCX)

S5 Table Quality assessment of each study included. (DOCX)

S6 Table Summary of methodological procedures of each study included (those sharing no details are not shown in the list). H&E stain, hematoxylin and eosin stain; NR, not reported; SAT, sugar absorption test; PAS stain, periodic acid–Schiff stain; SD, standard deviation.
(DOCX)

S7 Table Summary of dietary assessment of each study included (those sharing no details are not shown in the list). GFD, gluten-free diet; NR, not reported; NDG diet, do detectable gluten diet.
(DOCX)

S1 File Forest plots of disappearance of villous atrophy (control Marsh 0–2 ratio). (DOCX)

S2 File Forest plot of patients with strict adherence concerning complete mucosal recovery (control Marsh 0 ratio). (DOCX)

S3 File Metaregressions. (DOCX)

S4 File Publication bias. (DOCX)

S5 File Prisma checklist. (DOC)
