# Supplementary material for: Younger age at diagnosis predisposes to mucosal recovery in celiac disease on a gluten-free diet: A meta-analysis
Source: PLoS One. 2017 Nov 2;12(11):e0187526. doi: 10.1371/journal.pone.0187526 (PMC5695627; doi:10.1371/journal.pone.0187526)
Supplement: S1 Appendix — (PDF) [file pone.0187526.s001.pdf]

PROSPERO International prospective register of systematic reviews

Review title and timescale

1 Review title
Give the working title of the review. This must be in English. Ideally it should state succinctly the interventions or exposures being reviewed and the associated health or social problem being addressed in the review.
Mucosal recovery in celiac children and adults on gluten-free diet: a meta-analysis

2 Original language title
For reviews in languages other than English, this field should be used to enter the title in the language of the review. This will be displayed together with the English language title.

3 Anticipated or actual start date
Give the date when the systematic review commenced, or is expected to commence.
01/12/2016

4 Anticipated completion date
Give the date by which the review is expected to be completed.
01/02/2017

5 Stage of review at time of this submission
Indicate the stage of progress of the review by ticking the relevant boxes. Reviews that have progressed beyond the point of completing data extraction at the time of initial registration are not eligible for inclusion in PROSPERO. This field should be updated when any amendments are made to a published record.

The review has not yet started ×

Review stage	Started	Completed
Preliminary searches	Yes	Yes
Piloting of the study selection process	Yes	Yes
Formal screening of search results against eligibility criteria	Yes	No
Data extraction	No	No
Risk of bias (quality) assessment	Yes	No
Data analysis	No	No

Provide any other relevant information about the stage of the review here.

Review team details

6 Named contact
The named contact acts as the guarantor for the accuracy of the information presented in the register record.
Bajor Judit

7 Named contact email
Enter the electronic mail address of the named contact.
bajor.judit8@gmail.com

8 Named contact address
Enter the full postal address for the named contact.
13 Ifjúság út, 7624 Pécs, Hungary

9 Named contact phone number
Enter the telephone number for the named contact, including international dialing code.
+36203425244

10 Organisational affiliation of the review
Full title of the organisational affiliations for this review, and website address if available. This field may be completed as 'None' if the review is not affiliated to any organisation.
1st Department of Medicine, School of Medicine, University of Pécs

Website address:
http://aok.pte.hu/en/egyseg/index/260

11 Review team members and their organisational affiliations
Give the title, first name and last name of all members of the team working directly on the review. Give the organisational affiliations of each member of the review team.

Title	First name	Last name	Affiliation
Mr	Zsolt	Szakács	School of Medicine, University of Pécs, Hungary
Mr	Péter	Mátrai	Institute of Bioanalysis, University of Pécs, Hungary
Professor	Péter	Hegyi	Institute for Translational Medicine, University of Pécs, Hungary
Dr	András	Garami	Institute for Translational Medicine, University of Pécs, Hungary
Dr	Imre	Szabó	1st Department of Medicine, School of Medicine, University of Pécs, Hungary
Dr	Áron	Vincze	1st Department of Medicine, School of Medicine, University of Pécs, Hungary
Dr	Márta	Balaskó	Institute for Translational Medicine, University of Pécs, Hungary
Dr	László	Czopf	1st Department of Medicine, School of Medicine, University of Pécs, Hungary
Dr	Katalin	Márta	Institute for Translational Medicine, University of Pécs, Hungary
Dr	Alexandra	Mikó	Institute for Translational Medicine, University of Pécs, Hungary
Dr	Dániel	Pécsi	Institute for Translational Medicine, University of Pécs, Hungary
Mr	Zoltán	Kiss	1st Department of Paediatrics, Semmelweis University, Hungary
Dr	Alexandra	Demcsák	Department of Pediatrics, University of Szeged, Hungary
Dr	Judit	Bajor	1st Department of Medicine, School of Medicine, University of Pécs, Hungary

12 Funding sources/sponsors
Give details of the individuals, organizations, groups or other legal entities who take responsibility for initiating, managing, sponsoring and/or financing the review. Any unique identification numbers assigned to the review by the individuals or bodies listed should be included.
No specific funders for the review.

13 Conflicts of interest
List any conditions that could lead to actual or perceived undue influence on judgements concerning the main topic investigated in the review.
Are there any actual or potential conflicts of interest?
None known

14 Collaborators
Give the name, affiliation and role of any individuals or organisations who are working on the review but who are not listed as review team members.

Title	First name	Last name	Organisation details

Review methods

15 Review question(s)
State the question(s) to be addressed / review objectives. Please complete a separate box for each question.

Is mucosal recovery more frequent in celiac children than in celiac adults keeping a gluten-free diet (GFD)?

Which factors do influence mucosal recovery rate?

16 Searches

Give details of the sources to be searched, and any restrictions (e.g. language or publication period). The full search strategy is not required, but may be supplied as a link or attachment.

PubMed and EMBASE databases will be searched. The following filters will be applied: human, English. Key: celiac AND disease AND ((mucosal AND healing) OR (mucosal AND recovery) OR (villous AND atrophy))

17 URL to search strategy

If you have one, give the link to your search strategy here. Alternatively you can e-mail this to PROSPERO and we will store and link to it.

I give permission for this file to be made publicly available

Yes

18 Condition or domain being studied

Give a short description of the disease, condition or healthcare domain being studied. This could include health and wellbeing outcomes.

Celiac disease is an immune-mediated systemic disorder. The consumption of gluten-containing cereals (rye, barley and wheat) triggers an abnormal T-cell mediated immune response. Today, the best therapeutic option is lifelong gluten-withdrawal (gluten-free diet, GFD). Although symptoms improve quickly and titers of celiac specific antibodies normalize on GFD, small intestinal mucosal damage can remain persistent. There are various recovery rates in the literature, yet for unknown reasons. Some authors suggest that persistent villous damage is a potential risk factor for further morbidities (such as osteoporosis and lymphoproliferative malignancies) and might have influence on mortality as well. However, recent guidelines do not recommend regular follow-up biopsies to monitor mucosal recovery.

19 Participants/population

Give summary criteria for the participants or populations being studied by the review. The preferred format includes details of both inclusion and exclusion criteria.

Celiac patients. We will include patients with celiac disease who are on gluten-free diet and at least one follow-up duodenal biopsy was taken. There is no need to present the histological results of the initial diagnostic biopsy, we will include patients diagnosed with HLA genotyping and/or celiac specific antibodies supporting the clinical celiac phenotype, according to the recent guidelines. It is not required to provide detailed information on the initial diagnostic process, statement about diagnosis is acceptable. We will include children and adults if data are reported selectively within the study. We will exclude studies where insufficient data are provided or no clear statement is made which allow us to distinguish the data of children and adults. Studies defining the age of adults as 0.05, we will reject our null hypothesis.

29 Analysis of subgroups or subsets

Give any planned exploration of subgroups or subsets within the review. 'None planned' is a valid response if no subgroup analyses are planned.

We plan to carry out the following subgroup analyses: patients with 12-month-long GFD and studies inquiring about adherence to GFD.

Review general information

30 Type and method of review

Select the type of review and the review method from the drop down list.

Prospective meta-analysis (PMA), Systematic review

31 Language

Select the language(s) in which the review is being written and will be made available, from the drop down list. Use the control key to select more than one language.

English

Will a summary/abstract be made available in English?

Yes

32 Country

Select the country in which the review is being carried out from the drop down list. For multi-national collaborations select all the countries involved. Use the control key to select more than one country.
Hungary

33 Other registration details
Give the name of any organisation where the systematic review title or protocol is registered together with any unique identification number assigned. If extracted data will be stored and made available through a repository such as the Systematic Review Data Repository (SRDR), details and a link should be included here.

34 Reference and/or URL for published protocol
Give the citation for the published protocol, if there is one.
Give the link to the published protocol, if there is one. This may be to an external site or to a protocol deposited with CRD in pdf format.

I give permission for this file to be made publicly available
Yes

35 Dissemination plans
Give brief details of plans for communicating essential messages from the review to the appropriate audiences.
Do you intend to publish the review on completion?
Yes

36 Keywords
Give words or phrases that best describe the review. (One word per box, create a new box for each term)
mucosal healing

mucosal recovery

celiac disease

children

adults

gluten-free diet

follow-up biopsy

age

persistent villous atrophy

37 Details of any existing review of the same topic by the same authors
Give details of earlier versions of the systematic review if an update of an existing review is being registered, including full bibliographic reference if possible.

38 Current review status
Review status should be updated when the review is completed and when it is published.
Ongoing

39 Any additional information
Provide any further information the review team consider relevant to the registration of the review.

40 Details of final report/publication(s)
This field should be left empty until details of the completed review are available.

Give the full citation for the final report or publication of the systematic review.
Give the URL where available.
