# Supplementary material for: Younger age at diagnosis predisposes to mucosal recovery in celiac disease on a gluten-free diet: A meta-analysis
Source: PLoS One. 2017 Nov 2;12(11):e0187526. doi: 10.1371/journal.pone.0187526 (PMC5695627; doi:10.1371/journal.pone.0187526)
Supplement: S2 Appendix — (PDF) [file pone.0187526.s002.pdf]

S2 APPENDIX

Search strategy in EMBASE

#1	celiac
#2	#1 AND disease
#3	#1 AND patients
#4	#2 NOT (celiac AND trunk)
#5	healing
#6	recovery
#7	mucosal AND (#5 OR #6)
#8	villous AND atrophy
#9	persistent AND #8
#10	follow-up
#11	#7 OR #8
#12	#2 AND #11

Searchkey in EMBASE
celiac AND ('disease'/exp OR disease) AND (mucosal AND ('healing'/exp OR healing OR 'recovery'/exp OR recovery) OR (villous AND ('atrophy'/exp OR atrophy)))

Searchkey in PUBMED
celiac[All Fields] AND ("disease"[MeSH Terms] OR "disease"[All Fields]) AND ((("mucous membrane"[MeSH Terms] OR ("mucous"[All Fields] AND "membrane"[All Fields]) OR "mucous membrane"[All Fields] OR "mucosal"[All Fields]) AND (("wound healing"[MeSH Terms] OR ("wound"[All Fields] AND "healing"[All Fields]) OR "wound healing"[All Fields] OR "healing"[All Fields]) OR recovery[All Fields])) OR (villous[All Fields] AND ("atrophy"[MeSH Terms] OR "atrophy"[All Fields])))

Searchkey in Cochrane Trials and Web of Science
celiac AND disease AND ((mucosal AND healing) OR (mucosal AND recovery) OR (villous AND atrophy))

Data extraction

Numeric and texted data were collected as follows.

Publication:

- first author, year and journal
- country
- recruitment period
- study design (prospective or retrospective)

Data for statistical analyses:

- number of participants in the eligible group(s)
- number of males in the eligible group(s) or male/total ratio
- age at diagnosis (years)
- duration of gluten-free diet (months)
- histological classification (with definitions if provided)
- initial histology
- follow-up histology

Data for methodological quality assessment:

- details on sampling procedure
- details on sample preparation
- details on histological assessment and inter-observer agreement
- details on dietary assessment (tool, timing and executor)
- duration of gluten-free diet (months)
- selection criteria
- initial histology
- data on follow-up (drop-out)
