# Supplementary material for: Younger age at diagnosis predisposes to mucosal recovery in celiac disease on a gluten-free diet: A meta-analysis
Source: PLoS One. 2017 Nov 2;12(11):e0187526. doi: 10.1371/journal.pone.0187526 (PMC5695627; doi:10.1371/journal.pone.0187526)
Supplement: S1 Table — (DOCX) [file pone.0187526.s003.docx]

| **Study** | **Definitions** |
| --- | --- |
| Bannister, 2014 | Mucosal healing: < Marsh grade 3 |
| Vécsei, 2014 | Mucosal healing: < Marsh grade 2 |
| Rubio-Tapia, 2010 | Mucosal recovery: villous to crypt ratio ≥ 3:1, the number of intraepithelial lymphocytosis was not a criteria |
| Lanzini, 2009 | Normalization: Marsh 0; remission: reconstitution of normal villous architecture and persistent intraepithelial lymphocytosis |
| Newnham, 2015 | Mucosal remission: Marsh grade 0; mucosal response: Marsh grades 0-1 |
| Wahab, 2002 | Histologic remission: < Marsh grade 3 |
| Galli, 2014 | Complete histological recovery: Marsh grade 0 |
| Bhasin, 2010 | Complete recovery: Marsh grade 0 |
| Martini, 2002 | Mucosal recovery: Marsh grade 0 |
| Lidums, 2011 | Histological response: < Marsh grade 3 |
| Lichtwark, 2014 | Mucosal remission: Marsh grade 0; mucosal response: Marsh grade 1 |
| Vivas, 2009 | Mucosal recovery: < Marsh grade 3 |
| Hære, 2016 | Mucosal recovery: < Marsh grade 3; mucosal healing: Marsh 0 |
| Tuire, 2012 | Mucosal recovery: Marsh grades 1-2 |
| Pekki, 2015 | Complete mucosal recovery: villous to crypt ratio < 2; mucosal recovery: villous to crypt ratio ≥ 2 |
| Hutchinson, 2010 | Complete histopathological recovery: Marsh grade 0 |
| Tursi, 2006 | Complete recovery: villous to crypt ratio ≥ 4:1 |
| Sharkey, 2013 | Histologic recovery: < Marsh grade 3 or Corazza Grade A |
