# Supplementary material for: Younger age at diagnosis predisposes to mucosal recovery in celiac disease on a gluten-free diet: A meta-analysis
Source: PLoS One. 2017 Nov 2;12(11):e0187526. doi: 10.1371/journal.pone.0187526 (PMC5695627; doi:10.1371/journal.pone.0187526)
Supplement: S2 Table — (DOCX) [file pone.0187526.s004.docx]

|  | **Adapted Newcastle-Ottawa Scale** | **High-quality items carrying a low risk of bias**  **(1 star)** | **Low-quality items carrying a high^*^ or an unknown^#^ risk of bias**  **(0 stars)** |
| --- | --- | --- | --- |
| **Selection** | **Item 1:** Representativeness of the initial study population | Non-selected group(s) of celiac patients. Exclusion of conditions increasing the risk of upper endoscopy (e.g., terminal disease, active bleeding, pregnancy), patients with conditions carrying differential diagnostic risk (i.e., flat mucosa), selection upon age and/or duration of gluten-free diet and/or diagnostic histology are assessed as low risk. | Selected group(s) of celiac patients^*^ or no description^#^ |
|  | **Item 2:** Ascertainment of gluten-free diet | Dietary questionnaire and/or structured interview about gluten-free diet | Food diary only (equal to written self-report)^*^ and/or measurement of celiac specific antibodies^*^ or no description^#^ |
|  | **Item 3:** Demonstration that outcome of interest was not present at start of study | Marsh grade 3 or equal histopathology at diagnosis in all participants | Anything less than Marsh grade 3 or equal histopathology at diagnosis in any participants^*^ or no description^#^ |
| **Outcome** | **Item 4:** Assessment of follow-up histology | Histological assessment in a blinded fashion (to the clinical status) on follow-up | Unblinded fashion of histological assessment on follow-up^*^ or no description^#^ |
|  | **Item 5:** Was follow-up long enough for outcomes to occur? | ≥12-month gluten-free diet for all participants prior to the follow-up biopsy | <12-month gluten-free diet for any participant(s) prior to the follow-up biopsy^*^ or no description^#^ |
|  | **Item 6:** Adequacy of follow-up | Complete follow-up or subjects lost to follow up unlikely to introduce bias: description provided of those lost (e.g., no consent given for upper endoscopy, death) | No description of the lost^*^ |
