# Supplementary material for: Younger age at diagnosis predisposes to mucosal recovery in celiac disease on a gluten-free diet: A meta-analysis
Source: PLoS One. 2017 Nov 2;12(11):e0187526. doi: 10.1371/journal.pone.0187526 (PMC5695627; doi:10.1371/journal.pone.0187526)
Supplement: S3 Table — NR, not reported; *, mean; #, median; s, same follow-up period for all participants. (DOCX) [file pone.0187526.s005.docx]

| **Study (first author, year, and subgroups)** | | | **Number of patients (n)** | **Sex ratio (male/total)** | **Age at diagnosis (years)** | **GFD length (months)** | **Histology (ratio)** | | |
| --- | --- | --- | --- | --- | --- | --- | --- | --- | --- |
|  |  |  |  |  |  |  | **Initial villous atrophy** | **Complete mucosal recovery** | **Disappearance of villous atrophy** |
| Annibale, 2001 | | | 7 | 0.14 | NR | 12^s^ | 1 | 0.43 | 0.57 |
| Assiri, 2008 | | | 12 | NR | NR | NR | NR | NR | 0.5 |
| Bannister, 2014 | | | 150 | 0.43 | 7.5^#^ | 16.8^#^ | NR | 0.83 | 0.95 |
| Bardella, 2007 | | Children | 135 | 0.39 | 1^#^ | 24^#^ | 1 | 0.74 | NR |
|  |  | Adults | 114 | 0.28 | 33^#^ | 24^#^ | 1 | 0.18 | 0.37 |
| Baudon, 2005 | | | 25 | NR | NR | NR | NR | NR | 0.52 |
| Bhasin, 2010 | | Children | 34 | NR | NR | NR | NR | 0.5 | NR |
|  |  | Total | 56 | 0.54 | 20.16^*^ | 23.76^*^ | 1 | 0.46 | 0.5 |
| Biagi, 2012 | | | 141 | 0.23 | NR | 27^#^ | NR | NR | 0.86 |
| Cammarota, 2007 | | | 62 | 0.16 | NR | 11.27^*^ | NR | NR | 0.6 |
| Capristo, 2009 | | | 26 | 0.35 | NR | 14.2^*^ | 1 | 0.62 | 1 |
| Carroccio, 2008 | | | 69 | 0.17 | NR | 76.1^*^ | NR | 0 | 0.34 |
| Caruso, 2014 | | | 13 | 0.08 | NR | 84^#^ | NR | NR | 0.85 |
| Casella, 2012 | | | 800 | NR | NR | NR | NR | 0.04 | 0.81 |
| Chaisemartin, 2015 | | | 100 | 0.22 | NR | 54^#^ | NR | 0.18 | 0.32 |
| Ciacci, 2002 | | | 390 | 0.23 | 27.9^*^ | 82.68^*^ | 0.95 | 0.44 | 0.76 |
| Ciacci, 2005 | | | 110 | 0.34 | 0.9^*^ | 188.4^*^ | NR | NR | 0.65 |
| Congdon, 1981 | | | 10 | NR | NR | NR | NR | 0.5 | NR |
| Cuoco, 1999 | | | 23 | 0.3 | 32^*^ | 12^s^ | NR | NR | 0.57 |
| Dickey, 2000 | | | 53 | 0.26 | 51^*^ | 12^s^ | 1 | 0.38 | 0.39 |
| Donaldson, 2008 | | | 16 | NR | NR | NR | NR | 0.13 | 1 |
| Duerksen, 2010 | | | 21 | 0.14 | NR | 120^*^ | NR | NR | 0.71 |
| Elli, 2015 | | | 69 | 0.24 | 39^*^ | 48^*^ | 1 | 0.29 | 0.47 |
| Galli, 2014 | | | 65 | 0.28 | 38^#^ | 12^s^ | 1 | 0.54 | 0.67 |
| Ghazzawi, 2014 | | | 40 | 0.35 | 8.5^*^ | 24^*^ | 0.90 | 0.63 | 0.84 |
| Gorgun, 2009 | | | 7 | NR | NR | 13.1^*^ | 1 | 0.57 | 0.86 |
| Günther, 2010 | | | 30 | 0.3 | NR | NR | NR | 0.1 | 0.23 |
| Hære, 2016 | | | 127 | 0.38 | NR | 111.6* | NR | 0.81 | 0.94 |
| Hopper, 2008 | | | 48 | 0.31 | NR | NR | NR | 0.44 | 0.67 |
| Hutchinson, 2010 | | | 284 | 0.29 | 44.6^#^ | 19.2 | NR | 0.39 | NR |
| Karinen, 2006 | | | 96 | 0.41 | 43.1^*^ | 12^s^ | NR | NR | 0.34 |
| Kaukinen, 2002 | | | 87 | 0.28 | NR | 12^#^ | NR | 0.33 | 0.69 |
| Kemppainen, 1998 | | | 34 | NR | NR | 12^s^ | NR | NR | 0.09 |
| Koskinen, 2010 | | | 105 | 0.33 | NR | 96^#^ | NR | NR | 0.98 |
| Lanzini, 2009 | Marsh classification | | 465 | 0.23 | 31^#^ | 16^#^ | NR | 0.08 | 0.8 |
|  | Marsh-Oberhuber classification | | 321 | NR | NR | NR | 0.90 | 0.02 | 0.87 |
| Lebwohl, 2013 | | | 7648 | 0.37 | 28.4^*^ | 15.6^#^ | NR | NR | 0.57 |
| Lee, 2003 | | | 39 | 0.37 | NR | 102^*^ | NR | NR | 0.21 |
| Lichtwark, 2014 | | | 10 | NR | NR | 12^s^ | NR | 0.4 | 0.9 |
| Lidums, 2011 | | | 12 | 0.33 | 44^*^ | 12^s^ | 1 | 0.33 | 0.59 |
| Martini, 2002 | | | 101 | 0.22 | 37^#^ | 12^*^ | NR | 0.12 | NR |
| McMillan, 2001 | | | 36 | 0.28 | NR | 12^*^ | 1 | 0.19 | 0.39 |
| Newnham, 2016 | | | 52 | 0.21 | 42^*^ | 12^s^ | 0.96 | 0.37 | 0.54 |
| O’Keeffe, 2001 | | | 12 | 0.25 | NR | 36^*^ | NR | NR | 0.5 |
| Pekki, 2015 | | | 263 | 0.32 | 45^#^ | 12^s^ | NR | NR | 0.65 |
| Raivio, 2006 | | | 91 | 0.33 | NR | 36^#^ | NR | NR | 0.97 |
| Rubio-Tapia, 2010 | | | 241 | 0.27 | 47^#^ | NR | NR | 0.34 | NR |
| Selby, 1999 | | | 89 | 0.18 | 38.9^*^ | 99.4^*^ | NR | NR | 0.57 |
| Sharkey, 2013 | | | 391 | NR | NR | 11^#^ | NR | 0.26 | 0.57 |
| Shmerling, 1986 | | | 24 | NR | 1.2^#^ | 46.08^#^ | NR | NR | 1 |
| Sjöberg, 2014 | | | 13 | 0.38 | 4.23^*^ | 13.1^*^ | NR | 0.92 | 0.92 |
| Tuire, 2012 | | | 177 | 0.27 | NR | NR | NR | 0.42 | NR |
| Tursi, 2006 | | | 42 | 0.31 | 32.7^#^ | 24^s^ | 0.81 | 0.6 | 0.88 |
| Uil, 1996 | Children | | 8 | NR | 5^#^ | 16^*^ | NR | 0.5 | 1 |
|  | Adults | | 9 | NR | 48^#^ | 9^*^ | NR | 0.22 | 0.56 |
| Vahedi, 2003 | | | 95 | 0.26 | 41^#^ | 75^#^ | NR | NR | 0.34 |
| Valdimarsson, 2000 | | | 98 | NR | NR | 12^s^ | NR | NR | 0.78 |
| Vécsei, 2009 | | | 27 | 0.44 | 44.52^*^ | 40^#^ | NR | NR | 0.48 |
| Vécsei, 2014 | | | 53 | 0.38 | NR | 26.4^#^ | 0.89 | 0.87 | 0.9 |
| Vivas, 2009 | | | 40 | NR | NR | NR | NR | NR | 0.75 |
| Volta, 2008 | | | 19 | NR | NR | 12^s^ | NR | 0.16 | 0.47 |
| Wahab, 2001 | | | 26 | 0.42 | NR | NR | NR | NR | 0.58 |
| Wahab, 2002 | Children | | 25 | 0.32 | NR | NR | NR | NR | 1 |
|  | Total | | 158 | 0.28 | NR | NR | 1 | 0.41 | 0.84 |
| Yachha, 2007 | | | 25 | 0.56 | 8.3^*^ | 14.88^*^ | 1 | 0.04 | 0.04 |
| Zanini, 2012 | | | 60 | NR | NR | NR | NR | 0.02 | 0.8 |

| **Study (first author, year, and subgroups)** | | | **Patients with good/strict adherence** | | |
| --- | --- | --- | --- | --- | --- |
|  |  |  | **Number of participants(n)** | **Histology (ratio)** | |
|  |  |  |  | **Complete mucosal recovery** | **Disappearance of villous atrophy** |
| Annibale, 2001 | | | NR | - | - |
| Assiri, 2008 | | | NR | - | - |
| Bannister, 2014 | | | NR | - | - |
| Bardella, 2007 | | Children | 135 | 0.74 | NR |
|  |  | Adults | 114 | 0.18 | 0.37 |
| Baudon, 2005 | | | NR | - | - |
| Bhasin, 2010 | | Children | NR | - | - |
|  |  | Total | NR | - | - |
| Biagi, 2012 | | | 116 | NR | 0.93 |
| Cammarota, 2007 | | | NR | - | - |
| Capristo, 2009 | | | NR | - | - |
| Carroccio, 2008 | | | 69 | 0 | 0.34 |
| Caruso, 2014 | | | 13 | NR | 0.85 |
| Casella, 2012 | | | NR | - | - |
| Chaisemartin, 2015 | | | NR | - | - |
| Ciacci, 2002 | | | 166 | 0.93 | 0.99 |
| Ciacci, 2005 | | | NR | - | - |
| Congdon, 1981 | | | 10 | NR | 0.80 |
| Cuoco, 1999 | | | 13 | NR | 0.85 |
| Dickey, 2000 | | | 53 | 0.38 | 0.39 |
| Donaldson, 2008 | | | NR | - | - |
| Duerksen, 2010 | | | NR | - | - |
| Elli, 2015 | | | NR | - | - |
| Galli, 2014 | | | 53 | 0.66 | 0.81 |
| Ghazzawi, 2014 | | | NR | - | - |
| Gorgun, 2009 | | | 7 | 0.57 | 0.86 |
| Günther, 2010 | | | NR | - | - |
| Hære, 2016 | | | NR | - | - |
| Hopper, 2008 | | | NR | - | - |
| Hutchinson, 2010 | | | NR | - | - |
| Karinen, 2006 | | | NR | - | - |
| Kaukinen, 2002 | | | 76 | 0.36 | 0.71 |
| Kemppainen, 1998 | | | NR | - | - |
| Koskinen, 2010 | | | NR | - | - |
| Lanzini, 2009 | Marsh classification | | NR | - | - |
|  | Marsh-Oberhuber classification | | NR | - | - |
| Lebwohl, 2013 | | | NR | - | - |
| Lee, 2003 | | | NR | - | - |
| Lichtwark, 2014 | | | 10 | 0.40 | 0.90 |
| Lidums, 2011 | | | NR | - | - |
| Martini, 2002 | | | NR | - | - |
| McMillan, 2001 | | | NR | - | - |
| Newnham, 2016 | | | 45 | 0.49 | 0.85 |
| O’Keeffe, 2001 | | | NR | - | - |
| Pekki, 2015 | | | NR | - | - |
| Raivio, 2006 | | | 91 | NR | 0.97 |
| Rubio-Tapia, 2010 | | | NR | - | - |
| Selby, 1999 | | | 50 | NR | 0.60 |
| Sharkey, 2013 | | | NR | - | - |
| Shmerling, 1986 | | | NR | - | - |
| Sjöberg, 2014 | | | NR | - | - |
| Tuire, 2012 | | | 177 | 0.42 | NR |
| Tursi, 2006 | | | 39 | 0.64 | 0.92 |
| Uil, 1996 | Children | | NR | - | - |
|  | Adults | | NR | - | - |
| Vahedi, 2003 | | | 40 | NR | 0.65 |
| Valdimarsson, 2000 | | | NR | - | - |
| Vécsei, 2009 | | | NR | - | - |
| Vécsei, 2014 | | | NR | - | - |
| Vivas, 2009 | | | NR | - | - |
| Volta, 2008 | | | NR | - | - |
| Wahab, 2001 | | | NR | - | - |
| Wahab, 2002 | Children | | NR | - | - |
|  | Total | | NR | - | - |
| Yachha, 2007 | | | 25 | 0.04 | 0.04 |
| Zanini, 2012 | | | NR | - | - |
