# Supplementary material for: Younger age at diagnosis predisposes to mucosal recovery in celiac disease on a gluten-free diet: A meta-analysis
Source: PLoS One. 2017 Nov 2;12(11):e0187526. doi: 10.1371/journal.pone.0187526 (PMC5695627; doi:10.1371/journal.pone.0187526)
Supplement: S4 Table — (DOCX) [file pone.0187526.s006.docx]

| **Study** | **Rationale for exclusion** |
| --- | --- |
| Lebwohl, 2013 | identical initial cohorts from the same register |
| Lebwohl, 2015 |  |
| Lebwohl, 2015 |  |
| Lebwohl, 2014 |  |
| Lebwohl, 2014 |  |
| Bonamico, 2004 | no gluten-free diet initiated |
| Rostami, 1999 |  |
| Cogulu, 2003 |  |
| Ertekin, 2010 |  |
| Hawamdeh, 2016 |  |
| Dickey, 2008 | not eligible initial groups (untreated, persistent villous atrophy only, mucosal recovery only) |
| Valdimarsson, 1994 |  |
| Zanini, 2016 |  |
| Kaukinen, 2007 |  |
| Ejderhamn, 1992 |  |
| Murray, 2016 |  |
| Dipper, 2009 | overlapping biopsy results (multiple biopsies in the same data pool) |
| Dissenayake, 1974 | no access to the article |
| Hussain, 1999 |  |
| Kilander, 2009 | no control biopsy taken/lacking data about control mucosal histology and/or patients |
| Kurppa, 2010 |  |
| Magliocca, 1996 |  |
| Rodrigo-Saez, 2011 |  |
| Taha, 2005 |  |
| Troncone, 1995 |  |
| Abrams, 2004 |  |
| Achour, 2010 |  |
| Brar, 2006 |  |
| Ioannou, 2011 |  |
| Murray, 2008 |  |
| Poddar, 2002 |  |
| Volta, 2014 |  |
| Singh, 2015 |  |
| Lancaster-Smith, 1976 | uncertain diagnosis (e.g., only tenuous/potential celiac patients at study entry)/miscellaneous celiac and non-celiac patients |
| Tosco, 2011 |  |
| Agardh, 2004 |  |
| Rostami, 1999 |  |
| Crenn, 2003 |  |
| Kotze, 2016 |  |
| Shmidt, 2013 |  |
| Newnham, 2014 | updated version available and included |
| Lundin, 2003 | case report |
