# Supplementary material for: Younger age at diagnosis predisposes to mucosal recovery in celiac disease on a gluten-free diet: A meta-analysis
Source: PLoS One. 2017 Nov 2;12(11):e0187526. doi: 10.1371/journal.pone.0187526 (PMC5695627; doi:10.1371/journal.pone.0187526)
Supplement: S5 Table — (DOCX) [file pone.0187526.s007.docx]

|  | **Items** | | | | | | **Total** |
| --- | --- | --- | --- | --- | --- | --- | --- |
|  | **1** | **2** | **3** | **4** | **5** | **6** |  |
| Kaukinen, 2002 | 0 | 1 | 1 | 0 | 1 | 1 | **4** |
| Kemppainen, 1998 | 0 | 0 | 1 | 1 | 1 | 0 | **3** |
| Koskinen, 2010 | 1 | 0 | 0 | 1 | 1 | 1 | **4** |
| Lanzini, 2009 | 1 | 1 | 0 | 0 | 1 | 1 | **4** |
| Lebwohl, 2013 | 1 | 0 | 1 | 0 | 0 | 1 | **3** |
| Lee, 2003 | 0 | 0 | 1 | 0 | 1 | 1 | **3** |
| Lichtwark, 2014 | 0 | 1 | 0 | 1 | 1 | 0 | **3** |
| Lidums, 2011 | 1 | 0 | 1 | 0 | 1 | 1 | **4** |
| Martini, 2002 | 1 | 0 | 0 | 0 | 0 | 1 | **2** |
| McMillan, 2001 | 1 | 0 | 1 | 1 | 0 | 1 | **4** |
| Newnham, 2016 | 1 | 1 | 0 | 1 | 1 | 0 | **4** |
| Pekki, 2015 | 1 | 0 | 1 | 0 | 1 | 1 | **4** |
| Rubio-Tapia, 2010 | 1 | 1 | 1 | 0 | 0 | 1 | **4** |
| Selby, 1999 | 1 | 1 | 0 | 1 | 0 | 1 | **4** |
| Sharkey, 2013 | 1 | 1 | 1 | 0 | 0 | 1 | **4** |
| Shmerling, 1986 | 0 | 0 | 1 | 1 | 1 | 1 | **4** |
| Tuire, 2012 | 0 | 1 | 0 | 1 | 1 | 1 | **4** |
| Tursi, 2006 | 1 | 1 | 0 | 1 | 1 | 1 | **5** |
| Uil, 1996 | 1 | 0 | 1 | 1 | 0 | 1 | **4** |
| Vahedi, 2003 | 0 | 1 | 1 | 0 | 1 | 1 | **4** |
| Valdimarsson, 2000 | 1 | 0 | 1 | 0 | 1 | 0 | **3** |
| Vécsei, 2009 | 0 | 0 | 0 | 0 | 1 | 1 | **2** |
| Vécsei, 2014 | 1 | 1 | 0 | 1 | 1 | 1 | **5** |
| Vivas, 2009 | 0 | 0 | 0 | 0 | 1 | 1 | **2** |
| Wahab, 2001 | 1 | 0 | 0 | 0 | 1 | 1 | **3** |
| Wahab, 2002 | 1 | 0 | 1 | 1 | 1 | 1 | **5** |
| Yachha, 2007 | 1 | 1 | 1 | 0 | 1 | 1 | **5** |

|  | **Items** | | | | | | **Total** |
| --- | --- | --- | --- | --- | --- | --- | --- |
|  | **1** | **2** | **3** | **4** | **5** | **6** |  |
| Annibale, 2001 | 0 | 0 | 1 | 0 | 1 | 1 | **3** |
| Assiri, 2008 | 1 | 0 | 1 | 0 | 0 | 0 | **2** |
| Bannister, 2014 | 1 | 1 | 1 | 1 | 1 | 1 | **6** |
| Bardella, 2007 | 0 | 1 | 1 | 0 | 1 | 1 | **4** |
| Baudon, 2005 | 1 | 1 | 1 | 0 | 1 | 0 | **4** |
| Bhasin, 2010 | 0 | 1 | 1 | 1 | 1 | 1 | **5** |
| Biagi, 2012 | 0 | 1 | 1 | 0 | 0 | 1 | **3** |
| Cammarota, 2007 | 0 | 0 | 0 | 1 | 1 | 1 | **3** |
| Capristo, 2009 | 0 | 0 | 1 | 0 | 1 | 1 | **3** |
| Carroccio, 2008 | 1 | 1 | 0 | 1 | 1 | 1 | **5** |
| Caruso, 2013 | 1 | 0 | 0 | 1 | 1 | 1 | **4** |
| Casella, 2012 | 1 | 1 | 0 | 0 | 1 | 0 | **3** |
| Chaisemartin, 2015 | 1 | 1 | 1 | 1 | 1 | 1 | **6** |
| Ciacci, 2002 | 0 | 1 | 0 | 0 | 1 | 1 | **3** |
| Ciacci, 2005 | 1 | 1 | 0 | 0 | 0 | 1 | **3** |
| Congdon, 1981 | 1 | 1 | 0 | 1 | 1 | 1 | **5** |
| Dickey, 2000 | 0 | 1 | 1 | 1 | 1 | 1 | **5** |
| Donaldson, 2008 | 0 | 0 | 1 | 0 | 0 | 0 | **1** |
| Elli, 2015 | 0 | 0 | 1 | 1 | 1 | 1 | **4** |
| Galli, 2014 | 1 | 1 | 1 | 1 | 1 | 1 | **6** |
| Ghazzawi, 2014 | 1 | 0 | 0 | 0 | 0 | 1 | **2** |
| Gorgun, 2009 | 1 | 0 | 1 | 0 | 0 | 1 | **3** |
| Günther, 2010 | 0 | 1 | 0 | 1 | 1 | 1 | **4** |
| Hære, 2016 | 1 | 1 | 1 | 1 | 1 | 1 | **6** |
| Hopper, 2008 | 1 | 0 | 1 | 0 | 1 | 1 | **4** |
| Hutchinson, 2010 | 1 | 0 | 0 | 0 | 0 | 1 | **2** |
| Karinen, 2006 | 0 | 0 | 1 | 1 | 1 | 0 | **3** |
