# Supplementary material for: Younger age at diagnosis predisposes to mucosal recovery in celiac disease on a gluten-free diet: A meta-analysis
Source: PLoS One. 2017 Nov 2;12(11):e0187526. doi: 10.1371/journal.pone.0187526 (PMC5695627; doi:10.1371/journal.pone.0187526)
Supplement: S6 Table — H&E stain, hematoxylin and eosin stain; NR, not reported; SAT, sugar absorption test; PAS stain, periodic acid–Schiff stain; SD, standard deviation. (DOCX) [file pone.0187526.s008.docx]

| **Study** | **Duodenal Sampling procedure** | **Sample preparation** | **Histological classification** | **Histological assessment** |
| --- | --- | --- | --- | --- |
| Annibale, 2001 | 2 samples from the second part | Fixation in Bouin's solution for 4-8 hours at room temperature, 5-μm paraffin sections, H&E and PAS stains | Modified Marsh: complete resolution, mild abnormalities, partial, subtotal and total atrophy | NR |
| Bannister, 2014 | At least 4 samples (mean: 5, range: 3-10) including at least one from the duodenal cap | Immersion-fixation in formalin, embedding, perpendicular sectioning and the best-oriented areas were chosen for assessment | Marsh-Oberhuber | Two independent pediatric pathologists, blinded to clinical data, the most affected area was chosen |
| Bardella, 2007 | At least 4 samples, including the proximal and distal parts | Orientation on Millipore filter paper, fixation in 10% formalin, embedding in paraffin wax, 5-μm sections, H&E stain. Immunohistochemistry (anti-CD3). | Marsh-Oberhuber (IELs: <25/100) | NR |
| Bhasin, 2010 | At least 4 samples from the second part | Orientation on paper and fixation in 10% neutral formalin | Marsh (IEL: ≤30/100) | Two pathologists in a double-blinded fashion |
| Biagi, 2012 | NR | NR | Persistent atrophy (present or not present) after O'Mahony et al. | NR |
| Cammarota, 2007 | 4 samples from the distal part by the same skilled endoscopist | Orientation on filter, fixation in formalin, H&E stain | Marsh-Oberhuber | Multiple pathologists blinded to the clinical data, at least 4-6 crypt units were counted |
| Capristo, 2009 | NR | NR | Marsh-Oberhuber | NR |
| Carroccio, 2008 | At least 6 samples from the second part | Orientation on Millipore filter paper, fixation in 10% formalin, embedding in paraffin wax, H&E stain | Marsh-Oberhuber (IELs≤30/100) | Experienced pathologists blinded to the clinical data |
| Caruso, 2014 | 3 experienced endoscopists with an independent observer supervision by using standard forceps. 4 samples from the bulb, another 4 from the distal part from different quadrants | Orientation on tissue paper, fixation in formalin, embedding in paraffin | Corazza-Vilanacci | Experienced pathologists blinded to the clinical data |
| Casella, 2012 | At least 4 samples | Orientation on cellulose filters | Marsh-Oberhuber | NR |
| Chaisemartin, 2015 | At least 5 samples, 1 from the buld and 4 from the distal part | Embedding in paraffin wax | Marsh-Oberhuber (IELs: ≤30/100) | An experienced pathologist blinded |
| Ciacci, 2002 | 3 samples | NR | Marsh-Oberhuber (IELs: <40/100) | NR |
| Ciacci, 2005 | NR | Orientation on paper, fixation in formalin | Marsh-Oberhuber (IELs: ≤40/100) | NR |
| Congdon, 1981 | Pediatric capsule | NR | Normal to minor abnormalities, mild to moderate and severe partial atrophy, subtotal atrophy | An author blinded to dietary evaluation |
| Cuoco, 1998 | At least 5 samples beyond the Vatel-papilla | H&E stain | Atrophy, crypt hyperplasia, IELs | NR |
| Dickey, 2000 | 3 samples from the second part with standard forceps | Orientation on filter, fixation in formalin | Marsh and Rostami | An experienced pathologist blinded to the initial histology and laboratory data |
| Donaldson, 2008 | Taken by forceps | H&E stain | Marsh-Oberhuber | Assessed by board-certified pathologists, reviewed by a pediatric gastroenterologist |
| Duerksen, 2010 | At least 4 samples from the second part | Fixation in formalin, H&E stain | Modified Marsh (by Ravelli, 2005) | An investigator in a blinded fashion |
| Elli, 2015 | 4 samples | Orientation on adhesive filter paper, fixation in 10% buffered formalin, embedding in paraffin, 3-μm sections, H&E stain. Immunohistochemistry (anti-CD3) | Marsh-Oberhuber (IELs <25/100) | Independently by two expert pathologists blinded to the clinical status |
| Galli, 2014 | At least 4 samples from the second part with flexible video gastroscope by endoscopist blinded to the previous appearance | H&E stain. Immunohistochemistry (anti-CD3) | Marsh-Oberhuber (IELs: ≤30/100) | An expert pathologist |
| Ghazzawi 2014 | NR | NR | Modified Marsh by Antonioli 2003 (IELs: ≤25/100) | Pathologic reports were reviewed by an author |
| Gorgun, 2009 | Multiple samples from the bulb and distal part | Orientation on filter, fixation in 40g/L formaldehyde, embedding in paraffin, 4-μm sections, H&E stain | Marsh | NR |
| Günther, 2010 | Biopsies from multiple sites oriented with confocal endomicroscopy, an additional biopsy oriented with acrilflavine 0.05% stain | Fixation in buffered formalin, embedding in paraffin wax, 2-3-μm sections, H&E stain | Marsh-Oberhuber | Two pathologists, blinded to the endoscopic results |
| Hære, 2016 | 4 samples from the distal part | No orientation, fixation in 4% formaldehyde, H&E stain. Immunohistochemistry (anti-CD3) | Marsh Oberhuber (IELs ≤25/100) | Pathologists A (locally, not blinded) and B (reference, blinded to the clinical status) |
| Hopper, 2008 | 4 samples from the second part | Fixation in buffered formalin, embedding in paraffin wax, 3-μm sections, H&E stain | Marsh-Oberhuber | NR |
| Hutchinson, 2010 | NR | NR | modified Marsh grades | NR |
| Karinen, 2006 | NR | Fixation in 10% buffered formalin, van Gieson’s staining, orientation with dissecting microscope | Normal, partial and total atrophy | A pathologist blinded to the clinical status |
| Kaukinen, 2002 | From the distal part | H&E stain | Marsh | NR |
| Kemppainen, 1998 | 2 samples taken at 5-cm intervals starting in the bulb thereafter as far as possible with jumbo forceps | Fixation in 10% buffered formalin, van Gieson's staining, orientation with dissecting microscope | Normal, partial and total atrophy | A pathologist assessed blinded to the clinical state |
| Koskinen, 2010 | 6 samples with an adult-size Watson capsule from the proximal part with forceps | Frozen samples or embedding in paraffin wax, immunhistochemistry | Villous-crypt ratio was measured as detailed in the text | Blinded to the clinical data |
| Lanzini, 2009 | At least 4 samples from the distal part | Orientation on filter paper. Immunohistochemistry (anti-CD3). | Marsh and Marsh-Oberhuber (IELs ≤25/100) | The worst histological lesion was chosen |
| Lebwohl, 2013 | NR | NR | Marsh | Local pathologists |
| Lee, 2003 | At least 6 samples from the second part with standard forceps | No orientation, H&E stain | Normal, partial and total atrophy | An expert pathologist |
| Lichtwark, 2014 | 2 samples from the first part and at least 4 ones from the second part | NR | Marsh score | An experienced pathologist blinded to the clinical data |
| Lidums, 2011 | NR | NR | Marsh-Oberhuber (IELs≤40/100) | NR |
| Martini, 2002 | NR | NR | Marsh | NR |
| McMillan, 2001 | 3 samples from the distal part with standard forceps | Orientation on filter, fixation in formalin | Marsh and Rostami (IELs: ≤30/100) | A single person blinded to serology |
| Newnham, 2015 | At least 4 samples from the second part | NR | Marsh | An experienced pathologist blinded to patient information and baseline histology |
| Pekki, 2015 | At least 6 samples from the distal part | Orientation, 5-μm sections, H&E stain. Immunohistochemistry (anti-CD3). | Villous-crypt ratio was calculated (IELs: ≤30/100) | At least 3 well-oriented crypt units were assessed |
| Raivio, 2006 | Taken on upper endoscopy or with Watson capsule | H&E stain | Villous-crypt ratio was calculated | NR |
| Rubio-Tapia , 2010 | 4-6 samples from the second part | NR | Marsh-Oberhuber (IELs: <40/100) | The worst histological lesion was chosen |
| Selby, 1999 | 3 samples from the third part | NR | Villous-crypt ratio was calculated | Blinded to the clinical status |
| Sharkey, 2013 | 4 samples from the second part from different quadrants | NR | Marsh-Oberhuber | Dedicated gastrointestinal pathologists |
| Shmerling, 1986 | NR | NR | After Shmerling et al. 1970 | A person blinded to the clinical data with stereo-microscopy |
| Sjöberg, 2014 | NR | H&E stain | Marsh | NR |
| Tuire, 2012 | 6 samples from the distal part | As they did in a previous article | Marsh (IELs: <25/100) | The same investigator blinded to the clinical status |
| Tursi, 2006 | At least 6 samples from the second part with a disposable biopsy forceps with spike, additional 4 samples from the bulb, if appeared micronodular at endoscopy | H&E stain | Marsh (IELs: ≤30/100) | 2 expert pathologists blinded to the suspected diagnosis and endoscopic findings |
| Uil, 1996 | NR | NR | normal, crypt hyperplasia with IELs, partial and (sub)total atrophy | An author scored the biopsies blinded to SAT results |
| Vahedi, 2003 | 3-6 samples (mean: 4) | NR | Marsh-Oberhuber | NR |
| Valdimarsson, 2000 | NR | NR | Alexander | NR |
| Vécsei 2009 | 4 samples from the distal part and 2 from the bulb | Fixation in 4% phosphate-buffered formalin | Marsh-Oberhuber | NR |
| Vécsei, 2014 | 6 samples: 4 ones from the second part, another 2 ones from the bulb | NR | Marsh-Oberhuber (IELs: <30/100) | Two experienced pathologists blinded to the patients and indication of biopsy |
| Vivas, 2009 | NR | NR | Marsh-Oberhuber | NR |
| Volta, 2008 | NR | NR | Marsh-Oberhuber | NR |
| Wahab, 2001 | Taken with endoscopic guided Crosby capsule | NR | Marsh-Rostami (IELs: ≤30/100) | NR |
| Wahab, 2002 | Samples from the distal part with endoscopic guided Crosby capsule, with or without large forceps | NR | Marsh (IELs: ≤30/100) | Two of the authors blinded to the clinical status |
| Yachta, 2007 | 4-6 samples from the second and third part | Fixation in 10% buffered formalin, orientation, 3-μm sections, H&E stain | Marsh | A single pathologist |
| Zanini, 2012 | At least 4 samples from the distal part | Orientation on cellulose filter | Marsh | A single pathologist |
