# Supplementary material for: Younger age at diagnosis predisposes to mucosal recovery in celiac disease on a gluten-free diet: A meta-analysis
Source: PLoS One. 2017 Nov 2;12(11):e0187526. doi: 10.1371/journal.pone.0187526 (PMC5695627; doi:10.1371/journal.pone.0187526)
Supplement: S7 Table — GFD, gluten-free diet; NR, not reported; NDG diet, do detectable gluten diet. (DOCX) [file pone.0187526.s009.docx]

| **Study** | **Dietary assessment** | |
| --- | --- | --- |
|  | **Tool** | **By whom and when?** |
| Annibale, 2001 | Celiac-specific antibodies | NR |
| Bannister, 2014 | Biagi's questionnaire from 0 (no diet) to 4 scores (total gluten-free) | Non-dietetic personnel via telephone or in person during clinical consultations or on endoscopy |
| Bardella, 2007 | Dietary interview | Dietitian |
| Baudon, 2005 | Dietary survey: the amount of ingested gluten per day was estimated | NR |
| Bhasin, 2010 | Dietary interview | NR |
| Biagi, 2012 | Biagi's questionnaire from 0 (no diet) to 4 scores (total gluten-free) | Just before clinical re-evaluation (i.e., follow-up biopsy) |
| Carroccio, 2008 | Dietary interview: optimal or not optimal | Regularly on 6-12-month basis by trained dieticians |
| Casella, 2012 | Likert scale from 1 (no dietary digression) to 4 (no diet at all) | Attending physician at follow-up |
| Chaisemartin, 2015 | Dietary interview: adequate or significant gluten exposure | Dietitian |
| Ciacci, 2002 | Knowledge Rating Scale (self-rating scale), Dietetic Restriction Compliance (visual analogue scale) as permanent, transient or never on GFD | NR |
| Ciacci, 2002 | Dietary interview, assessed as very low, low or good | Trained physician |
| Congdon, 1981 | Dietary interview, assessed as strict, infrequent lapses or inadequate diet | Dietitian |
| Cuoco, 1998 | Dietary questionnaire | NR |
| Dickey, 2000 | Dietary interview | At 12-month visit by a hospital dietician |
| Duerksen, 2010 | 3-day food diary, random check by phone (no gluten, trace amount, or major transgression) | Dietitian |
| Galli, 2014 | Biagi's questionnaire from 0 (no diet) to 4 scores (total gluten-free), assessed as adequate or inadequate | After 1 year on clinical visit |
| Ghazzawi, 2014 | NR | Experienced dietitian |
| Gorgun, 2009 | Self-reporting, assessed as strict or hypogluten diet | NR |
| Hære, 2016 | Celiac Disease Adherence Test with Likert scale, where a total score between 7-25 was calculated, higher scores (>17) indicate poor adherence | NR |
| Hutchinson, 2010 | Self-reporting, assessed as poor or good | At follow-up visits |
| Kaukinen, 2002 | Dietary interview and 3-day food diary assessed as strict (zero-tolerance) or any dietary transgressions | By dietitian |
| Kemppainen, 1998 | Food records | NR |
| Lanzini, 2009 | Likert scale graded from 1 (no dietary digression) to 4 (no diet at all) | At the time of control biopsy |
| Lee, 2003 | NR | Physician familiar with the GFD |
| Lichtwark, 2014 | 7-day food diary (complemented with face-to-face consultations but only if concerns of transgressions raised), assessed as excellent, good, fair or poor | At week 12 and 52, by an expert dietician |
| Newnham, 2015 | Dietary interview, 7-day food diary entries as excellent, good, fair or non-compliant | Dietitian at each follow up |
| Pekki, 2015 | No data on tool, assessed as adherent or not (zero-tolerance) | After 1 year by dietician |
| Rubio-Tapia , 2010 | Dietary interview, graded from 1 (total gluten-free) to 5 (non-compliant), then compiled as good, moderate or poor | Experienced dietitian |
| Selby, 1999 | Dietary interview , questionnaire and 1-week food diary assessed as Codex GFD or NDG-GFD | NR |
| Sharkey, 2013 | Dietary interview and 1-2-week food diary (if non-compliance is suspicious) | Multiple times during follow-up both clinician and dietician |
| Sjöberg, 2014 | Food diary, celiac-specific serology | Dietitian |
| Tuire, 2012 | Dietary interview and 4-day food diary | By trained dietitian |
| Tursi, 2006 | Dietary interview, assessed as optimal, good, fair or poor | At the time of endoscopy |
| Vahedi, 2003 | Dietary interview of the previous 2 months (semiquantitative Food Frequency Questionnaire), assessed in numbers of transgression per month and expressed in grams | A single skilled dietitian |
| Vécsei, 2009 | No data on tool, assessed as keeping or not keeping (at least one transgression per month) | Physician at follow-up |
| Vécsei, 2014 | Dietary interview | NR |
| Volta, 2008 | Dietary questionnaire (strict and low compliers) | By trained dietitian |
| Yachta, 2007 | Antibody testing and dietary interview | At the time or within 6 months of follow-up biopsy |
