# Supplementary material for: Younger age at diagnosis predisposes to mucosal recovery in celiac disease on a gluten-free diet: A meta-analysis
Source: PLoS One. 2017 Nov 2;12(11):e0187526. doi: 10.1371/journal.pone.0187526 (PMC5695627; doi:10.1371/journal.pone.0187526)
Supplement: S1 File — (DOCX) [file pone.0187526.s010.docx]

**Supplementary File 1. Forest plots of disappearance of villous atrophy (control Marsh 0-2 ratio)**

Forest plot displaying control Marsh 0-2 ratios of the included studies

Forest plot displaying control Marsh 0-2 ratios of children-to-adults comparison.

Forest plot displaying control Marsh 0-2 ratios of studies/groups with strict adherence.

Forest plot displaying control Marsh 0-2 ratios after 12^-^month gluten-free diet.
