# Supplementary material for: Younger age at diagnosis predisposes to mucosal recovery in celiac disease on a gluten-free diet: A meta-analysis
Source: PLoS One. 2017 Nov 2;12(11):e0187526. doi: 10.1371/journal.pone.0187526 (PMC5695627; doi:10.1371/journal.pone.0187526)
Supplement: S2 File — (DOCX) [file pone.0187526.s011.docx]

**Supplementary File 2. Forest plot of patients with strict adherence concerning complete mucosal recovery (control Marsh 0 ratio).**
