# Supplementary material for: Younger age at diagnosis predisposes to mucosal recovery in celiac disease on a gluten-free diet: A meta-analysis
Source: PLoS One. 2017 Nov 2;12(11):e0187526. doi: 10.1371/journal.pone.0187526 (PMC5695627; doi:10.1371/journal.pone.0187526)
Supplement: S3 File — (DOCX) [file pone.0187526.s012.docx]

**Supplementary File 3. Metaregressions**

Regression analysis for the association between age at diagnosis (in years) and complete mucosal recovery (logit(Marsh 0 ratio)) or disappearance of villous atrophy (logit(Marsh 0-2 ratio)).

**p=0.05** **p<0.01**

coefficient: -0.03 coefficient: -0.03

Regression analysis for the association between duration of gluten-free diet (in months) and complete mucosal recovery (logit(Marsh 0 ratio)) or disappearance of villous atrophy (logit(Marsh 0-2 ratio)), all studies included.

**p=0.62** **p=0.86**

coefficient: 0.01 coefficient: 0.00

Regression analysis for the association between duration of gluten-free diet (in months) and complete mucosal recovery (logit(Marsh 0 ratio)) or disappearance of villous atrophy (logit(Marsh 0-2 ratio)), short follow-up included (less than about 2 years)

 **p=0.13** **p=0.18**

coefficient: 0.09 coefficient: 0.04

Regression analysis for the association between diagnostic histologic severity (diagnostic Marsh 3 ratio) and complete mucosal recovery (logit(Marsh 0 ratio)) or disappearance of villous atrophy (logit(Marsh 0-2 ratio))

**p<0.01** **p<0.01**

coefficient: -8.97 coefficient: -12.09

Regression analysis for the association between male gender (ratio) and complete mucosal recovery (logit(Marsh 0 ratio)) or disappearance of villous atrophy (logit(Marsh 0-2 ratio))

**p<0.01 p=0.87**

coefficient: 6.04 coefficient: 0.24

Regression analysis for the association between study quality (overall score calculated by the adapted Newcastle-Ottawa Scale) and complete mucosal recovery (logit(Marsh 0 ratio)) or disappearance of villous atrophy (logit(Marsh 0-2 ratio))

**p=0.08 p=0.85**

coefficient: 0.28 coefficient: 0.02
