# Supplementary material for: Younger age at diagnosis predisposes to mucosal recovery in celiac disease on a gluten-free diet: A meta-analysis
Source: PLoS One. 2017 Nov 2;12(11):e0187526. doi: 10.1371/journal.pone.0187526 (PMC5695627; doi:10.1371/journal.pone.0187526)
Supplement: S4 File — (DOCX) [file pone.0187526.s013.docx]

**Supplementary File 4. Publication bias**

Funnel plot for complete mucosal recovery ratio (pooled control Marsh 0 ratios)

**Egger’s regression:** intercept: -0.13, standard error: 1.39, **p=0.93**

Funnel plot for disappearance of villous atrophy ratio (pooled control Marsh 0-2 ratios)

**Egger’s regression:** intercept: 0.97, standard error: 0.60, **p=0.11**
